# Optimization of high-throughput 16S rRNA gene amplicon sequencing: an assessment of PCR pooling, mastermix use and contamination

**DOI:** 10.1099/mgen.0.001115

**Published:** 2023-10-16

**Authors:** Dinesh Aggarwal, Diana Rajan, Katherine L. Bellis, Emma Betteridge, Joe Brennan, Catarina de Sousa, CARRIAGE Study Team‡, Julian Parkhill, Sharon J. Peacock, Marcus C. de Goffau, Josef Wagner, Ewan M. Harrison

**Affiliations:** ^1^​ Department of Medicine, University of Cambridge, Cambridge, UK; ^2^​ Wellcome Sanger Institute, Hinxton, Cambridge, UK; ^3^​ Department of Veterinary Medicine, University of Cambridge, Cambridge, UK; ^4^​ Tytgat Institute for Liver and Intestinal Research, University of Amsterdam, Amsterdam, Netherlands; ^5^​ Department of Public Health and Primary Care, University of Cambridge, Cambridge, UK

**Keywords:** microbiome, nasal samples, 16S rRNA gene, laboratory protocol, PCR pooling, PCR drift, mastermix, contamination, efficiency

## Abstract

16S rRNA gene sequencing is widely used to characterize human and environmental microbiomes. Sequencing at scale facilitates better powered studies but is limited by cost and time. We identified two areas in our 16S rRNA gene library preparation protocol where modifications could provide efficiency gains, including (1) pooling of multiple PCR amplifications per sample to reduce PCR drift and (2) manual preparation of mastermix to reduce liquid handling. Using nasal samples from healthy human participants and a serially diluted mock microbial community, we compared alpha and beta diversity, and compositional abundance where the PCR amplification was conducted in triplicate, duplicate or as a single reaction, and where manually prepared or premixed mastermix was used. One hundred and fifty-eight 16S rRNA gene sequencing libraries were prepared, including a replicate experiment. Comparing PCR pooling strategies, we found no significant difference in high-quality read counts and alpha diversity, and beta diversity by Bray–Curtis index clustered by replicate on principal coordinate analysis (PCoA) and non-metric dimensional scaling (NMDS) analysis. Choice of mastermix had no significant impact on high-quality read and alpha diversity, and beta diversity by Bray–Curtis index clustered by replicate in PCoA and NMDS analysis. Importantly, we observed contamination and variability of rare species (<0.01 %) across replicate experiments; the majority of contaminants were accounted for by removal of species present at <0.1 %, or were linked to reagents (including a primer stock). We demonstrate no requirement for pooling of PCR amplifications or manual preparation of PCR mastermix, resulting in a more efficient 16S rRNA gene PCR protocol.

## Data Summary

All genomic data generated and/or analysed during the current study are available from the European Nucleotide Archive (ENA) under study accessions PRJEB64004 and PRJEB64269 (see Supplementary Material 1, available in the online version of this article for additional details). All Supplementary Materials can be found in the Figshare repository [1]. Data pertaining to samples obtained from the Wellcome Sanger Institute are for replication of analysis in this study or methods/protocol development only due to ethics constraints. Figshare link: https://doi.org/10.6084/m9.figshare.24188649.v1. [2]

**Impact Statement**

16S rRNA gene sequencing is a widely used method for characterizing human and environmental microbiomes that contribute to health and disease. An important advantage that 16S rRNA gene sequencing retains over shotgun metagenomic sequencing is the ability to sequence from low biomass samples and the lower cost of sequencing and computational resources required for analysis. Further streamlining the sequencing of 16S rRNA gene sequencing, with associated cost and time savings, without impacting on results would enable better powered microbiome studies. We systematically investigate the impact of the practice of pooling of PCR products (conducting 16S rRNA gene PCR in triplicate or duplicate) and the use of manually prepared mastermix or premixed mastermix; two important rate-limiting steps in 16S rRNA gene sequencing library preparation where modifications have the potential to provide a reduction in manual handling and cost savings, enabling 16S rRNA gene sequencing to be scaled up more effectively. We utilize low-biomass nasal samples and a mock microbial community; niches that are specifically lacking evidence for these comparisons. We demonstrate no significant difference in high-quality read count generation, diversity and compositionality from the comparison of PCR pooling strategies, and mastermix preparation. We reproduce these results in a replicate experiment to provide readers with confidence in applying these findings to their own protocols. Importantly, we find that contamination issues should actually be the main quality concern with low-biomass samples. Most contaminants could be linked to reagents, including an interesting finding of batch effects related to dual-indexed primer stock, and where contamination of negative controls was noted we display the additional benefit of using a mock microbial community as a positive control, which is also applicable to shotgun sequencing. These findings underline the necessity to carefully consider the choice of controls for low-biomass microbiome analyses and interpret findings relating to rare species with caution. The modified 16S rRNA gene library preparation protocol outlined in this study will enable the scaling of sequencing using liquid handling robotics, reduce manual handling steps and reduce the overall costs of sequencing, especially in low-biomass studies.

## Introduction

16S rRNA gene sequencing has been fundamental in characterizing human and environmental microbiomes that contribute to health and disease [3–6]. It remains a cost-effective and computationally less demanding alternative to shotgun metagenomic sequencing for taxonomic profiling [7, 8]. As the method is reference library independent, it can be employed for samples across hosts and environments, ranging from high to low microbial biomass in environmental and clinical settings, including those containing previously unidentified species. Furthermore, quantity both increases statistical power and enables the ascertainment of complex relationships between different species, including antagonistic relationships and (mutualistic) trophic networks [9, 10]. Further optimizing the cost-effectiveness of 16S rRNA gene sequencing so that thousands of samples can be readily studied would benefit research projects that are otherwise limited by funds and time. Efficient sequencing at scale requires greater efficiency of 16S rRNA gene PCR library preparation than is currently possible. This could be explored by identifying rate-limiting steps that can be streamlined, allowing the automation of laboratory processes. These are key to reducing operator time and costs, while increasing throughput, but without affecting the quality of results.

We identified and interrogated several steps that have limited evidence for their absolute necessity in our current 16S rRNA gene library preparation protocol (see Methods and Supplementary Material). These were as follows. (1) The need for multiple PCR amplifications per sample with subsequent pooling of products to (a) reduce PCR drift (i.e. the potential over-amplification of specific PCR products due to stochasticity in the PCR amplification) and (b) increase the overall yield of product, enabling the number of PCR cycles (which can also introduce bias) to be kept to a minimum [11–13]. (2) The use of a manually prepared mastermix, which increases liquid handling. Currently, preparing replicate PCR amplifications per sample prior to sequencing remains common. These are mostly in duplicates or triplicates, thereby increasing the total number of reactions prepared per experiment, which raises the risk of both environmental and sample-to-sample contamination, and error. Previous studies have evaluated the necessity of replicate PCR, but these have been limited by sample numbers or anatomical site [14–16] – none, to our knowledge, have evaluated nasal samples or used a diverse mock microbial community in serial dilutions (simulating samples with varying levels of microbial biomass).

Manual preparation of the mastermix solution in the 16S rRNA gene sequencing pipeline is not uncommon and the practice is often historical or arbitrary; it could be optimized to reduce manual handling. Available evidence on the direct comparison of mastermix solutions as a source of bias in 16S rRNA gene PCR sequencing is limited. Celis *et al.* examined three commercially available mastermix preparations and showed no difference, although this was evaluated for the specific purpose of developing a protocol for 16S rRNA gene sequencing of *in vitro* assemblies of gut communities [17]. Salter *et al.*, however, demonstrated that the ‘kitome’ becomes an increasingly important consideration with low-biomass samples using serial dilutions of *Salmonella bongori *, and showed extraction kit-specific contaminants when using shotgun metagenomic sequencing for nasopharyngeal samples [18]. Additionally, other studies comparing mastermix solutions have found commercially available preparations to be a potential source of contamination [19, 20], which can, however, be corrected for with appropriate controls [21]. Prior to a switch from a manually prepared PCR mastermix to a premixed mastermix, benchmarking of the premixed mastermix against the manual method was important to standardize and scale 16S rRNA gene sequencing protocols in our laboratory and, importantly, to evaluate whether a premixed mastermix results in a lower or higher risk of contamination.

Here, we evaluate the impact of pooling of PCR amplifications (single, duplicate and triplicate) and the use of manual versus premixed mastermix on the observed microbial diversity from human nasal samples and a mock microbial community.

## Methods

### Participants and samples

In this observational study, nasal samples taken from the anterior nares were obtained from healthy human participants from the community participating in the CARRIAGE study [22]. Additional healthy human nasal samples were collected anonymously from staff members of the Wellcome Sanger Institute between 8 August 2022 and 20 October 2022. A pre-extracted standard mock microbial community (ZymoBIOMICS Microbial Community DNA Standard II) was used [23]. This community contains a diverse set of eight bacterial strains and two fungal strains at varying DNA concentrations: *Listeria monocytogenes* (89.1 %), *Pseudomonas aeruginosa* (8.9 %), *Bacillus subtilis* (0.89 %), *Saccharomyces cerevisiae* (0.89 %), *Escherichia coli* (0.089 %), *Salmonella enterica* (0.089 %), *Lactobacillus fermentum* (0.0089 %), *Enterococcus faecalis* (0.00089 %), *Cryptococcus neoformans* (0.00089 %) and *Staphylococcus aureus* (0.000089 %). This allows for the examination of the impact of PCR pooling and mastermix choice on both highly abundant and rare species within a sample (rather than equivalent) and includes species with a range of genome sizes, rRNA gene copy numbers and G/C content. The mock community was serially diluted with nuclease-free water: undiluted, 1 : 10, 1 : 50 and 1 : 100.

### 16S rRNA gene PCR protocol

**Fig. 1.**
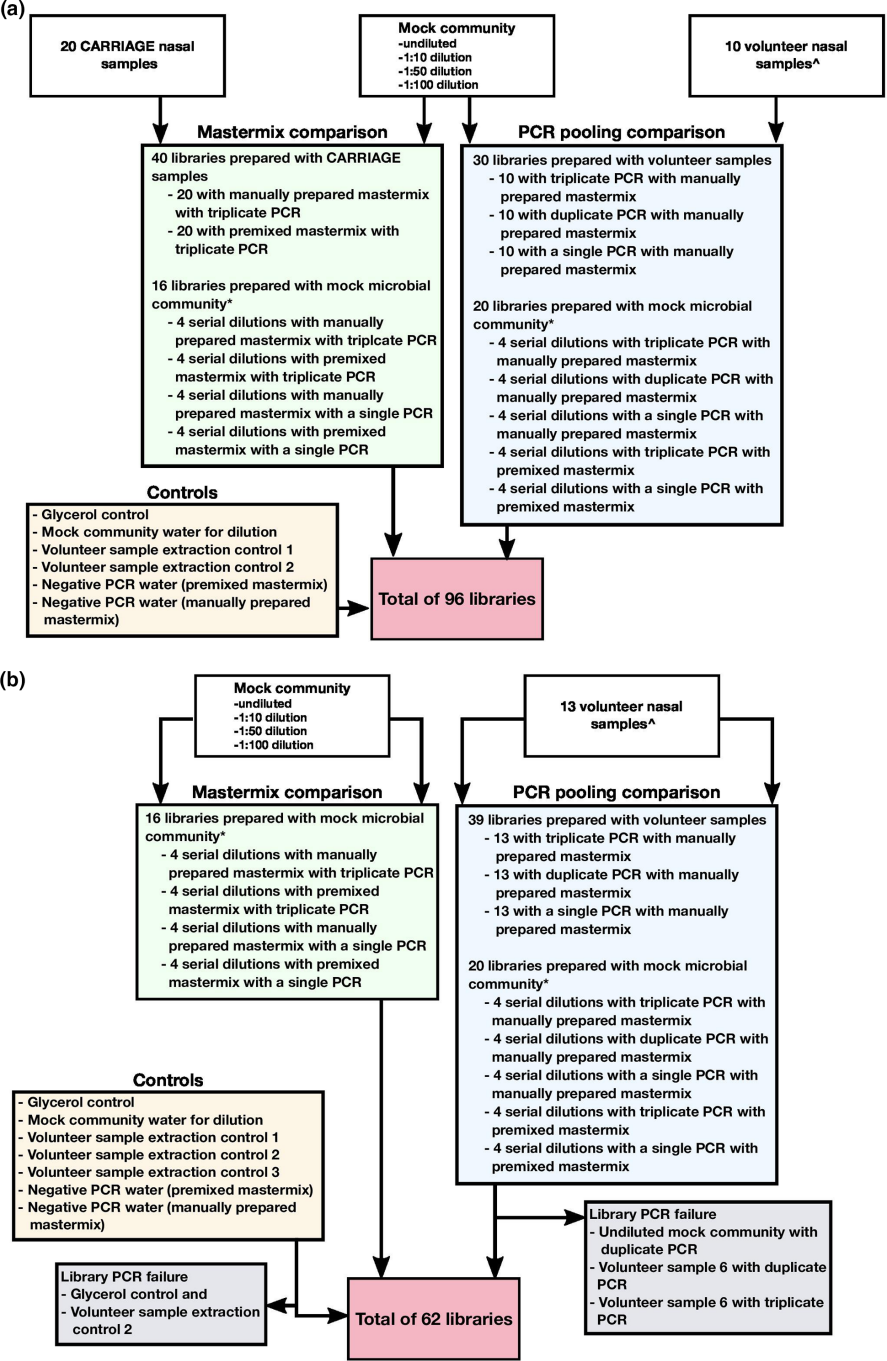
Samples and libraries prepared to compare PCR pooling and mastermix preparation. (**a**) Experiment 1. (**b**) Experiment 2 representing a replication study. In experiment 2, CARRIAGE samples were not available to evaluate the choice of mastermix preparation but the comparison was repeated with the mock community in serial dilutions. ^, nasal samples obtained from healthy participants from the Wellcome Sanger Institute. *, includes libraries that overlap in aims as the mock community in serial dilutions was evaluated with both manually prepared and premixed mastermix, and amplified with the PCR amplification in triplicate and as a single PCR amplification.

Total DNA was extracted from nasal sample transport medium using the MPBio MPure-12 instrument, (MPure Bacterial DNA kit, MP Biomedicals) with an additional mechanical lysis step (Lysing Matrix E, MP Biomedicals). Residual sample transport medium from nasal samples was stored at −70 °C in 33 % v/v glycerol before extraction. DNA was stored at −70 °C. PCR was performed to amplify bacterial 16S ribosomal gene regions using V1–V2-specific primers with attached sequencing adaptors and indexes (). PCR amplification mastermixes were either prepared manually using a Q5 High-Fidelity Polymerase kit (M0491, New England Biolabs) or with a premixed version using Q5 Hot Start High-Fidelity 2× Mastermix (M0494, New England Biolabs). PCR amplifications were set up either in triplicate (25 μl each), duplicate (40 μl each), or single (75 μl) reactions, depending on the conditions being tested, as outlined in Fig. 1. Duplicate and triplicate PCR products were pooled into single reactions per sample, and all samples were subsequently purified using an AMPure XP (Beckman Coulter) workflow at a ratio of 0.8×. Libraries were quantified using the AccuClear Ultra High Sensitivity dsDNA Quantitation kit (Biotium). Equimolar pools were created using a Biomek NX-8 liquid handler (Beckman Coulter). Negative controls included a sample extraction control, a PCR water control, an aliquot of the glycerol used for storage and an aliquot of the water used to dilute the mock community, whilst a positive control was represented by the mock microbial community. Contaminants were not removed from the analysis as these would provide additional information on sources of bias (if any observed) and would not be expected to impact on the primary study questions if seen at low levels, i.e. the impact of PCR pooling and PCR mastermix preparation on the microbial diversity and composition. We describe the contaminants in the Results section. Extraction and PCR protocols can be found in the Supplementary Methods and Results.

### PCR pooling and mastermix comparison experiments

#### Experiment 1

We used 10 nasal samples from healthy individuals at the Wellcome Sanger Institute and serial dilutions of the mock community to evaluate the output of 16S rRNA gene sequencing when the PCR amplification step was conducted in triplicate, duplicate or as a single reaction. These libraries were generated with the manually prepared mastermix. We used 20 nasal samples from the CARRIAGE study and serial dilutions of the mock community to evaluate the output of 16S rRNA gene sequencing when the PCR amplification mastermix was prepared manually or premixed. These libraries were prepared with the PCR amplification step conducted in triplicate. To evaluate the impact of combining both proposed optimizations, serial dilutions of the mock community were also prepared with the premixed mastermix and amplified with a single PCR amplification (Fig. 1a).

#### Experiment 2

To provide further confidence in the findings from this study, experiment 1 was repeated to evaluate the PCR amplification pooling strategy with an additional 3 nasal samples from participants from the Wellcome Sanger Institute, amounting to 13 nasal samples. Sufficient volumes of the samples from the CARRIAGE study were not available to repeat the second part of experiment 1 to evaluate the choice of mastermix preparation, but the comparison was repeated with serial dilutions of the mock community (Fig. 1b).

### DNA sequencing

Per experiment, an equimolar pool of PCR libraries was sequenced at the Sanger in-house sequencing facility, using Illumina MiSeq (300 bp paired-end reads, v3 reagent kit).

### Microbiome diversity analysis

The mothur MiSeq SOP was modified to process paired fastq files (MOTHUR wiki at http://www.mothur.org/wiki/MiSeq_SOP). The four poly(NNNN)s present in the adapter/primer sequences of contigs assembled with the ‘make.contigs’ command in mothur were trimmed in the PRINSEQ program, before the modified MiSeq SOP was resumed. The Silva bacterial database ‘silva.nr_v132.align’ was used to align quality-screened sequences. Chimaeras were removed using Uchime [24] and subsequently classified using the Silva reference database ‘silva.nr_v132.align’ and the Silva taxonomy database ‘silva.nr_v132.tax’, including the removal of Chloroplast, Mitochondria, unknown, Archaea and Eukaryota sequences. High-quality unique sequences were clustered with Oligotyping v2.1 [25] and assigned to NODES (similar to operational taxonomic units) with the ‘minimum entropy decomposition’ (MED) option. Taxonomic assignment was carried out with arb, using a customized silva SSU Ref database (NR99, release 132), where the majority of environmental and uncultured taxa were removed. In some instances, where a mismatch was observed within the taxanomic groups, the taxa of the NODE sequence was assigned with blast [26] (see Supplementary Methods and Results). The output was then combined in R (v4.2.1) into a phyloseq object for onward analysis.

### Diversity analysis

Microbial diversity and compositional analysis were conducted in R using the phyloseq (v1.40) [27] and vegan (v2.6–4) [28] packages. Alpha-diversity indices (Shannon’s, Simpson’s, Chao1 richness, observed richness and Fisher’s alpha) and beta-diversity indices (Bray–Curtis) were calculated on rarefied read counts. Sample microbial composition is represented with relative abundances. Principal coordinate analysis (PCoA) and non-metric dimensional scaling (NMDS) were carried out on the Bray–Curtis matrix to visualize the differences in sample diversity by experimental condition. For diversity, PCoA and NMDS analysis, high-quality reads from the operational taxonomic unit matrix were rarefied (9779 reads for experiment 1 and 38 794 reads for experiment 2) and then converted to percentage abundance in each sample.

### Data visualization and statistical analysis

Analysis was performed in Excel 2016 and R version 4.2.1. Figures were generated in R version 4.2.1 using ggplot2 (v3.4.0) and phyloseq (v1.40) [27]. Differences in read counts and alpha indices were evaluated with Mann–Whitney U and Kruskall–Wallis tests where appropriate. Kendall correlation co-efficient was used to examine the consistency of read counts and alpha indices between replicates from various study conditions where appropriate. PERMANOVA was used to estimate differences between Bray–Curtis distances observed by study groups with the vegan package (v2.6–4) [28]. A *P*-value <0.05 was considered statistically significant. This study complies with the STORM reporting guidelines for experimental/observational studies ().

## Results

### Pooling of PCR replicates

**Fig. 2.**
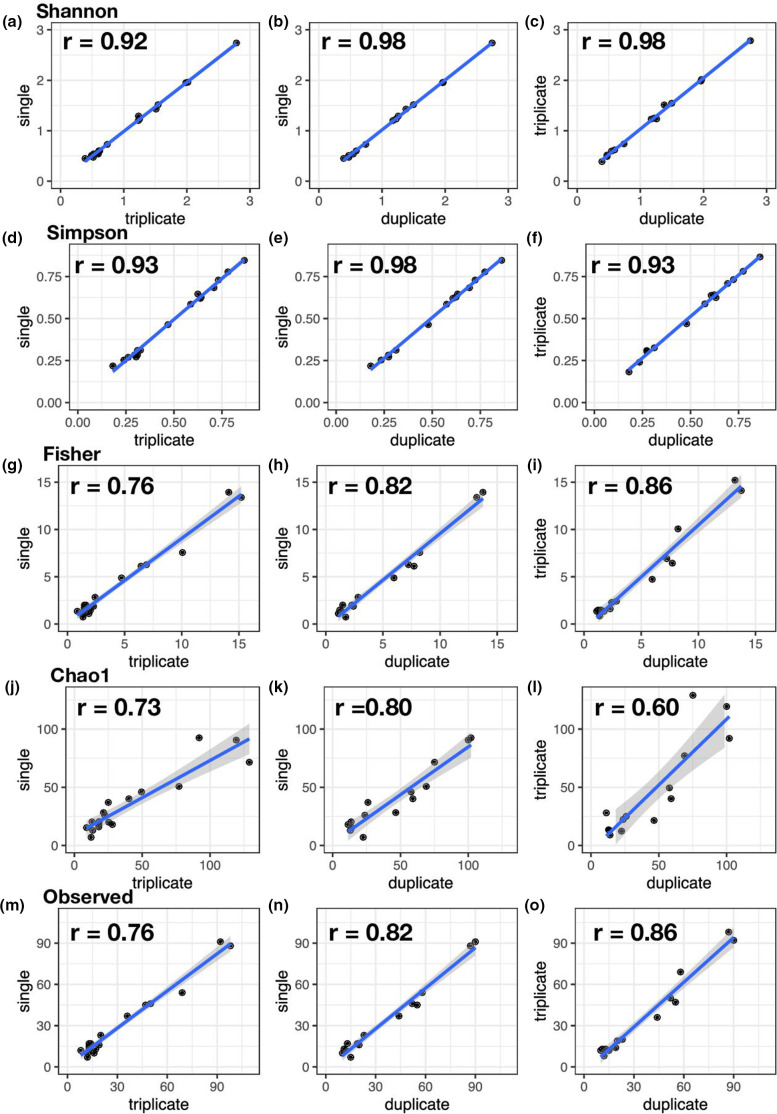
Correlation of alpha diversity indices by PCR pool after rarefication of high-quality sample reads with controls removed. Alpha indices represented include Shannon, Simpson, Fisher, Chao1 and observed richness (**a-o**). Pairwise Kendall’s rank correlation coefficient (**r**) is presented in the top-left of each plot. A strong correlation between PCR pools is observed by all alpha indices. A linear regression model is fitted to the observed values. Data presented are from experiment 1.

**Fig. 3.**
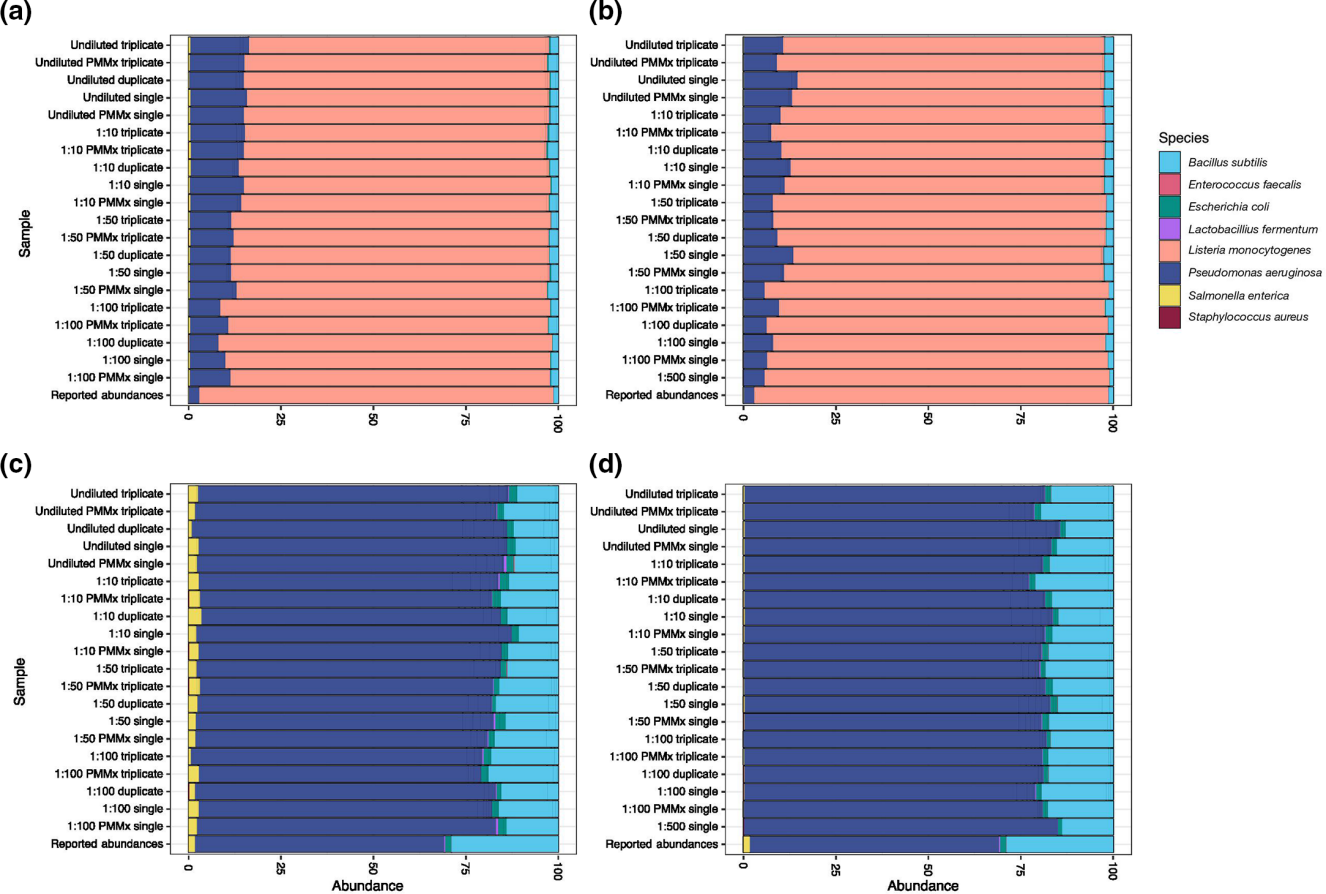
Relative abundance of species observed in the mock community replicates. (**a, c**) Experiment 1. (**b, d**) Experiment 2. Differences in abundance appear to be negligible by PCR pooling strategy and by mastermix. *Listeria* appears to be under-represented at lower levels of dilution (**a, b**). Relative abundance of species observed in the mock community replicates is shown with *Listeria* removed, to present amplification of rarer communities. Differences in the composition of ‘major’ species (>0.01 %) appear to be negligible by PCR pooling strategy and by mastermix (**c, d**). However, very-low-abundance species (<0.01 %) such as *Salmonella enterica* (yellow) and *Escherichia coli* (green), are more prone to variation between replicates, highlighting the need for careful validation of true lower abundance taxa through the analysis of replicates. Samples are grouped along the *y*-axis by mock community dilution. Further details on sample composition can be found in Table S3. PMMx, premixed mastermix.

**Fig. 4.**
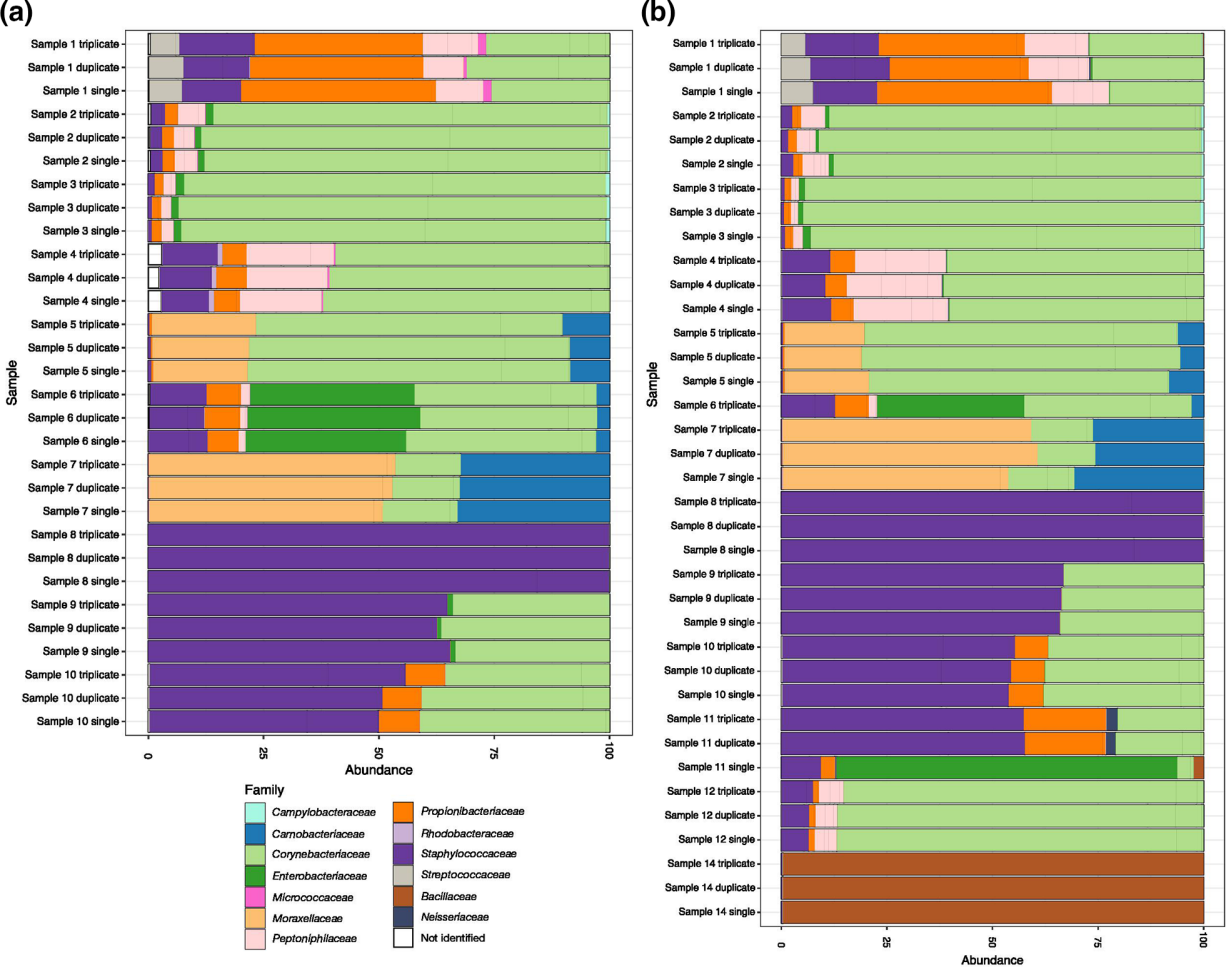
Relative abundance of taxa observed in the volunteer healthy nasal sample replicates by PCR pooling strategy. (**a**) Experiment 1. (**b**) Experiment 2. Relative abundance of taxa at the family taxonomic level appears consistent between different pooling strategies from samples relating to an individual participant nasal swab but varies appropriately between participants. Relative abundances are represented after rarefication of high-quality reads and removal of species at <1 % presence. Samples are grouped along the *y*-axis by healthy participant. NB heavy contamination with control species *Salmonella bongori* was observed in the PCR amplification performed in duplicate for sample 11 in experiment 2. Results from sample 6 are limited in experiment 2 due to PCR failure.

First, we investigated the impact of pooling PCR replicates on high-quality read count generation. The median read counts for libraries from experiment 1 (Fig. 1) were 146086, 146 727 and 128 310 from PCR amplifications including the microbial mock community and volunteer health nasal samples in triplicate, duplicate or as a single reaction, respectively (Fig. S1). Pairwise Mann–Whitney U test comparisons showed no significant difference in high-quality read counts generated from reactions in triplicate vs duplicate (*P*=0.54), triplicate vs single (*P*=0.58), or single vs duplicate (*P*=0.38). We then investigated variation in alpha diversity (measures of within-sample diversity) and beta diversity (measure of similarity or dissimilarity between two samples). We did not observe any significant difference between PCR pooling strategies using Kruskall–Wallis tests by Shannon, Simpson, Fisher, Chao1 and observed indices (Table S1 and Figs S2 and S3), and replicates from pair-wise PCR pool conditions showed a strong correlation by Kendall’s rank correlation coefficient (Fig. 2). Beta diversity was calculated by Bray–Curtis index clustered by replicate on examination of the PCoA and NMDS ordination plots, and did not significantly differ between PCR pooling strategies by PERMANOVA analysis *[F* (2) = 0.23, *P*=0.99]. As expected, the groups did differ by PERMANOVA analysis when compared by sample type i.e. mock vs healthy nasal sample [*F* (2) = 37.413, *P*<0.001] (Figs S4, S5 and S6). The relative abundance of microbial groups remained similar in technical replicates of each sample (including replicates of the same sample by the PCR pooling method prepared with the two mastermixes) (Figs 3 and 4). These findings were also consistent in experiment 2 (see Supplementary Methods and Results, Figs 3 and S4, S7–S12). When comparing experiments 1 and 2, we observed negligible differences in the composition of ‘major’ species (>0.01 %) by PCR pooling strategy and by mastermix (Fig. 3). However, very-low-abundance species (<0.01 %) such as *Salmonella enterica* and *Escherichia coli* are seen to be more prone to variation between replicates (samples in the same experiment and replicates between experiments), highlighting the need for careful validation of true lower abundance taxa through the analysis of replicates (Fig. 3). Overall, we show a similar alpha and beta diversity, and compositionality, of samples when 16S rRNA gene PCR amplification was performed in triplicate, duplicate or as a single reaction prior to sequencing.

### Mastermix preparation

**Fig. 5.**
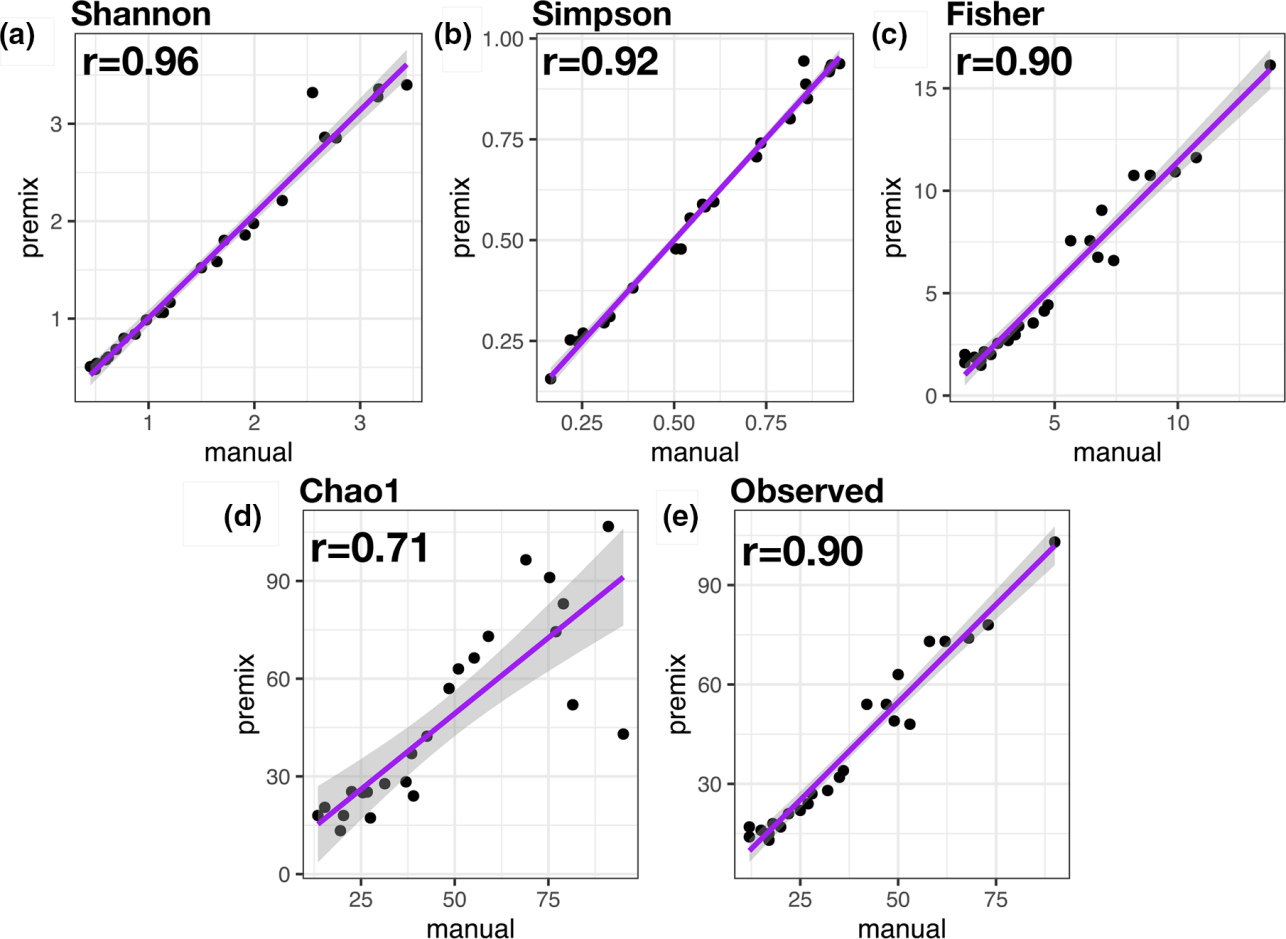
Correlation of alpha diversity indices by mastermix preparation (premixed vs manual) of high-quality sample reads. Alpha indices represented include Shannon, Simpson, Fisher, Chao1 and observed (**a–e**). Pairwise Kendall’s rank correlation coefficient (**r**) is presented in the top-left of each plot. A strong correlation between PCR pools is observed by all alpha indices. Alpha diversity calculated after rarefication of reads. A linear regression model is fitted to the observed values.

**Fig. 6.**
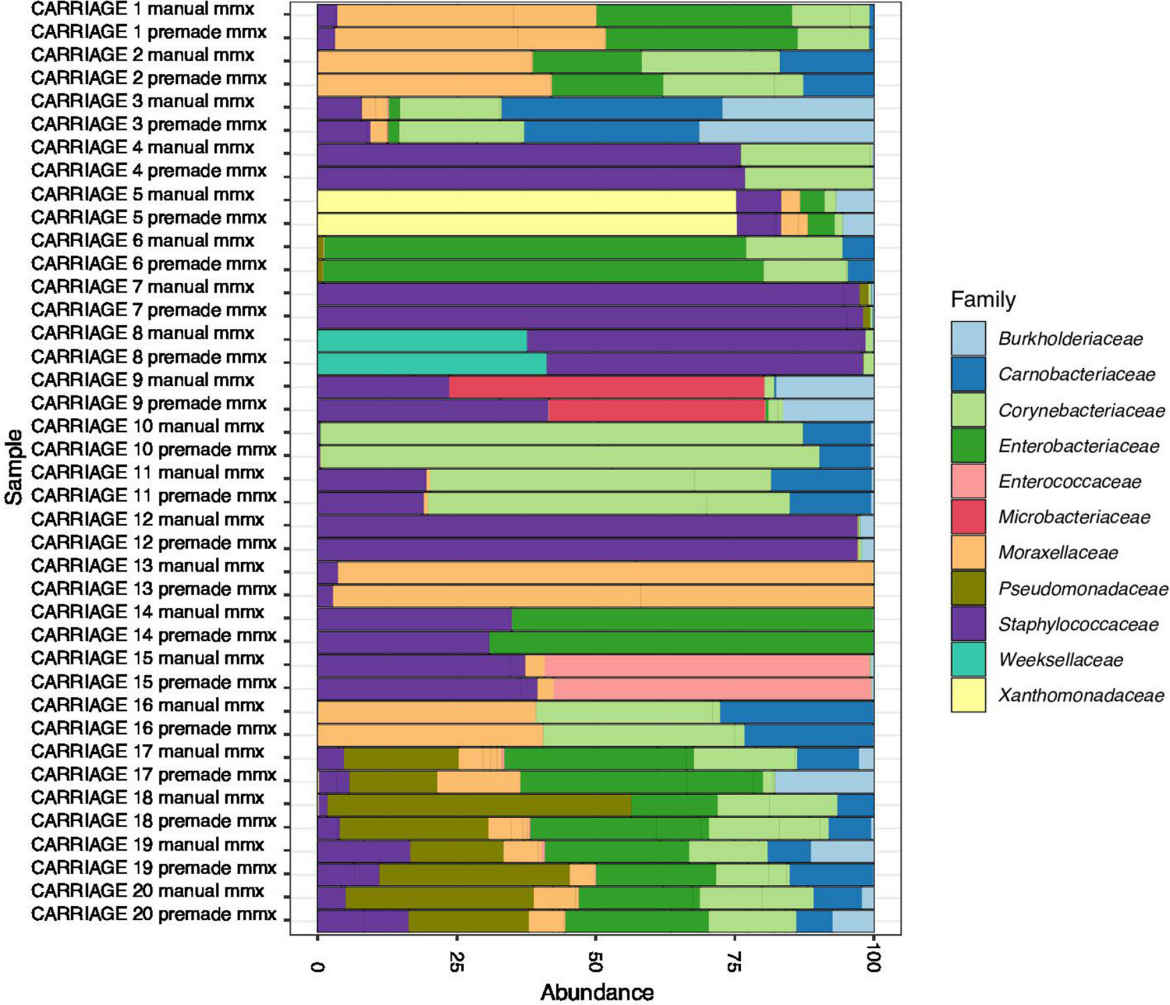
Relative abundance of species observed in the healthy nasal sample replicates by mastermix preparation. Relative abundance appears consistent between different mastermix preparations from samples relating to an individual participant nasal swab, but varies appropriately between healthy participants. Samples 17, 18, 19 and 20 include a *Salmonella bongori* spike at various dilutions (see Supplementary Data). Relative abundances are represented after rarefication of high-quality reads and species with <1 % abundance have been removed. Samples are grouped along the *y*-axis by healthy participant.

Next, we investigated the impact of mastermix preparation on high-quality read counts. After quality filtering of samples used to assess mastermixes (Fig. 1), the difference in read counts from samples with manually prepared mastermix (median=131658) or premixed mastermix (median=111890) by Mann–Whitney U test comparison did not reach statistical significance (*P*=0.05) (Figs S13 and S14). We then investigated variation in alpha diversity and beta diversity by mastermix preparation. Alpha diversity of replicates from manually prepared or premixed mastermix methods by Shannon, Simpson, Fisher, Chao1 and observed indices did not significantly differ by Mann–Whitney U comparison and demonstrated a strong correlation by Kendall’s rank correlation coefficient (Table S3, Figs 5 and S15). Beta diversity by Bray–Curtis index clustered by mastermix preparation replicate on examination of the PCoA and NMDS ordination plots, and did not differ significantly between mastermix preparations used by PERMANOVA analysis [*F* (2) = 0.25, *P*=1.00]. As expected, a significant difference was observed by sample type, i.e. mock vs healthy nasal sample by PERMANOVA analysis [*F* (2) = 28.862, *P*<0.001] (Figs S16 and S17). Further, the relative abundance of samples by all technical replicates (including various types of mastermix used) appeared to remain similar (Figs 3 and 6). In experiment 2, replicability was examined with the mock community serially diluted samples alone; composition of samples (Fig. 3), alpha diversity (Supplementary Methods and Results and Table S3) and beta diversity (Fig. S12) were seen to be similar between technical replicates. Overall, we show a similar alpha and beta diversity, and compositionality, of samples when 16S rRNA gene PCR gene amplification prior to sequencing is performed with a manually prepared mastermix or premixed mastermix.

### Contamination of samples

**Fig. 7.**
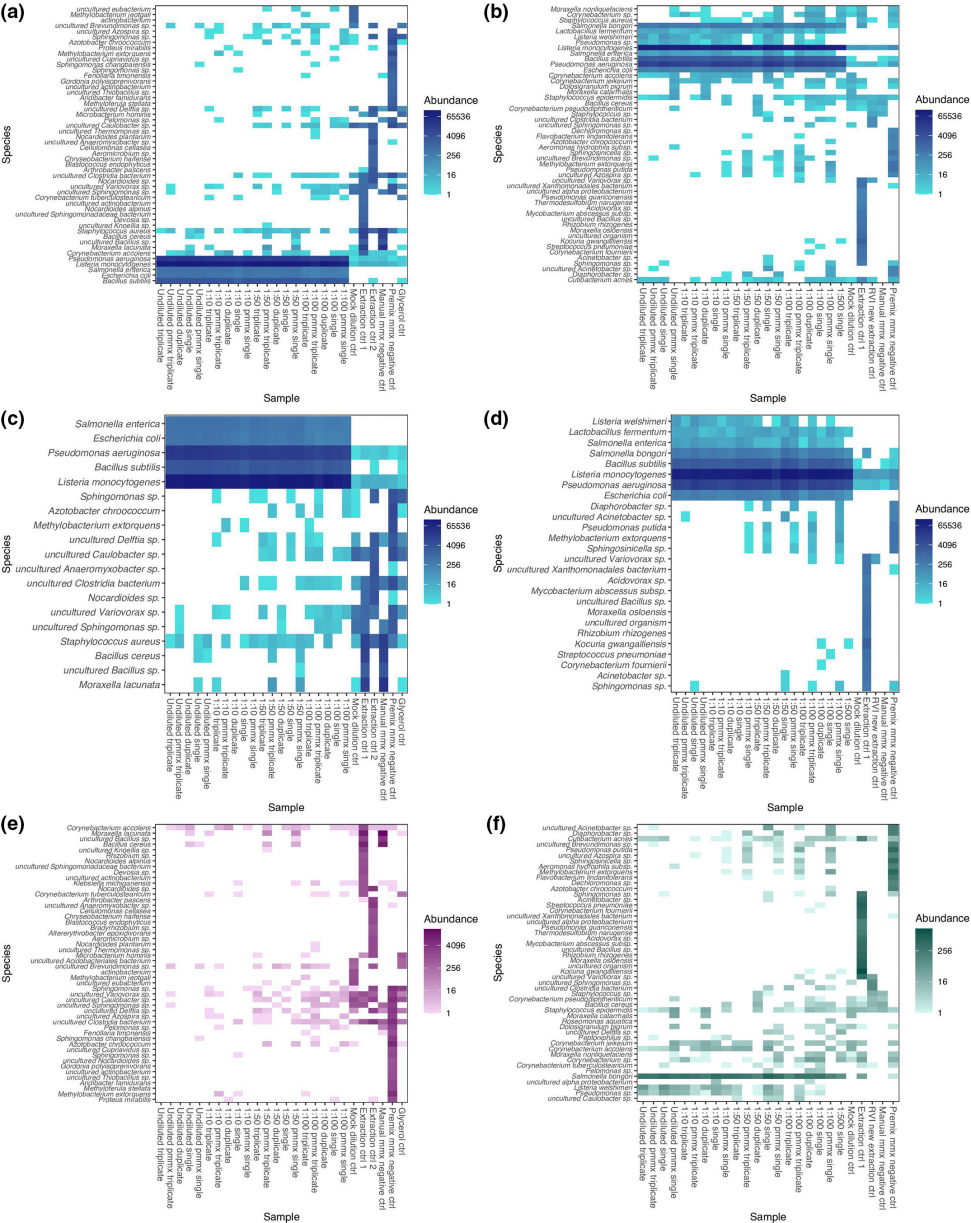
Heatmap of species abundances comparing controls and mock microbial community samples. Plots (a, c, e) correspond to experiment 1, whilst plots (b, d, f) correspond to experiment 2. Plots (a, b) demonstrate the highest 50 species by abundance. Plots (c, d) demonstrate the highest 50 species by abundance (if that many remain) after filtering for taxa that are >0.1 % abundance across all samples in experiment 1 and 0.01 % for experiment 2 (all contaminants were removed when filtering for species <0.1 %). Plots (e, f) demonstrate the contaminating species with the expected microbial communities removed. It is visible that contamination is occurring at low levels from controls to samples, and vice versa (e.g. mock community species seen in controls). Importantly, these are mostly accounted for when removing species of very low abundance and the remainder can be identified after systematic investigation of the controls. This highlights the need to include both negative and positive controls in microbiome studies of the low-biomass nasal niche. Read counts have undergone a log(4) transformation to better visualize low-abundance contaminants and their possible sources.

The controls were investigated systematically to understand possible sources of contamination. We utilized the expected mock microbial community composition to evaluate the nature and extent of contamination in experiments 1 and 2. We observed a degree of contamination of the controls that was most likely related to the kitome or laboratory equipment and/or environment in both experiments (Fig. 7). We noted contamination of our samples by the controls and vice versa but these could be accounted for as described below. After removing species present <0.1 % abundance, the majority of these contaminants were removed, demonstrating that these were largely present in very low concentrations (Fig. 7). Furthermore, by co-examining the most abundant contaminants of our controls and mock samples without the expected mock microbial community, we noted that the mock sample contaminant composition reflected the negative controls (therefore allowing identification of the source). For example, the contaminants of samples prepared with a premixed or manually prepared mastermix reflected their respective mastermix negative control (Fig. 7). As expected, contamination is seen to increase as the mock community dilution is increased (Fig. 7). When considering differences in the introduction of contaminants by PCR pooling strategy in experiment 1, we observed no significant difference in the read count of contaminants or the number of contaminating species using a Kruskall–Wallis test (*P*=0.69) (Fig. S18). Importantly, the most abundant species in the mock community samples reflected the dominant species as per the manufacturer’s guidance.

Specifically examining experiment 1 (Fig. 7), the water used for dilution of the mock community was included as a control, and contained contaminants consistent with the kitome. It also contained signals derived from species present in the mock community, but these had very low read counts (4, 1 and 10 reads of *L. monocytogenes *, *P. aeruginosa* and *S. aureus *, respectively). The glycerol for storage control (labelled Glycerol Ctrl) also contained the expected contaminants related to the kitome. Of note, the premixed and manually prepared mastermix PCR water, and the two swab extraction controls (labelled Extraction ctrl 1 and Extraction ctrl 2), were contaminated. The manually prepared mastermix and Extraction ctrl 1 were particularly contaminated with *Moraxella lacunata *, *S. aureus* and *Bacillus cereus *, and the premixed mastermix and Extraction ctrl 2 with expected kitome contaminants. Importantly, although contamination from controls to samples and vice versa was likely, these contaminants were in much lower abundances in our samples of interest (mock community and two cohorts of healthy nasal samples) and could be accounted for. The significant contaminants in the controls (such as *M. lacunata *) were seen in very low abundances in the samples of interest (Fig. 7), suggesting a lack of gross contamination of the experiment or its controls. This finding was confirmed with a lack of significant *M. lacunata* or *S. aureus* contamination observed in the controls from experiment 2 (Fig. 7) as the reagents were the same. Interestingly, we found a very low level of *M. lacunata* in all samples located on an isolated PCR plate row in experiment 1, and upon further investigation found all but one sample to be contaminated by low levels of *M. lacunata* in an isolated PCR plate row in experiment 2. This demonstrated a batch effect that was related to primer stock contamination – the only common factor (Fig. S19). Additionally, both the premixed mastermix and manually prepared mastermix were observed to contain contaminants at similar levels.

In experiment 2 (Fig. 7) low levels of contamination were again observed in, and from, our controls, from the kitome and from our samples. Specifically, we once again observed very minor contamination of the water used to dilute the mock community, with true mock community species (44, 6 and 1 reads of *L. monocytogenes *, *P. aeruginosa* and *B. subtilis *, respectively). The remaining controls were noted to include kitome contaminants in low abundances. Of note, in this experiment the most abundant contaminant across the mock community samples was *S. bongori. S. bongori* is used as a positive spike-in control in our laboratory and in this instance was seen to have contaminated a number of samples and was also observed in multiple negative controls (Fig. 7).

## Discussion

To our knowledge, we present the most complete assessment of the need for PCR pooling and the impact of mastermix preparation on the quality of 16S rRNA gene sequencing data generated from low-biomass nasal samples. We found that there was no significant difference in the estimations of high-quality read counts produced, alpha or beta diversity, or the composition of samples between 16S rRNA gene PCR amplifications carried out in triplicate, duplicate, or in a single reaction. We therefore demonstrate there is no need for PCR amplifications to be conducted in triplicate or duplicate to reduce PCR drift, at least in the settings of low biomass nasal microbiota analysis. Similarly, we observe no significant difference in outcomes from the use of manually prepared or premixed mastermix solutions. We analysed nasal swab samples from healthy human participants and a mock microbial community, and reproduced findings across experiments and technical replicates, providing further confidence in our results. Importantly, we observe that sample contamination (from the kitome, laboratory environment, or sample-to-sample) represents a greater source of uncertainty when examining low-biomass samples such as the anterior nares, especially in lower biomass samples, as the dilution series (Fig. 7) and experiments by Salter *et al.* [18] show, and the inclusion of both negative and positive controls is therefore vital.

Preparing replicate PCR amplifications adds significant time to the 16S rRNA gene sequencing workflow; it also increases the risk of both environmental and sample-to-sample contamination due to the additional number of liquid handling steps required. Manual preparation of PCR mastermix represents an extra manual handling step in the 16S rRNA gene sequencing process. Our findings therefore provide evidence to realize further efficiencies and streamlines this process. This is particularly relevant for large studies with thousands of samples but also reduces operator time for those conducting smaller scale studies. The nasal microbiome, and other low-biomass niches, have been vital in understanding human health and disease [29–31]; a leaner 16S rRNA gene sequencing protocol arising from this study (Supplementary Materials and protocols.io [32]) will facilitate scaling up of nasal and other microbiome studies in order to increase power and enable the *in silico* study of complex interactions between microbial groups. In addition, by sequencing hundreds or even thousands of samples, batch effect analyses can be applied more effectively to identify whether many of the signals are in fact contaminants [30].

Multiple reasons exist for biases in the PCR amplification process, and two important reasons include selection bias and drift. Selection bias entails the preferential annealing and amplification of templates due to the underlying properties of the genome, such as variations in GC content and a tendency for all templates to reach a 1 : 1 ratio in the PCR mixture, if run for enough cycles [11]. Drift refers to the stochastic nature of the PCR process. Polz *et al.* reported unexplained variability attributed to drift in the PCR products observed from PCR experiments using 16S rDNA templates derived from experimental conditions and suggested that the pooling of PCR replicates would reduce this bias [11]. These observations were in laboratory-generated microbial DNA mixtures and errors introduced through experimental handling could not be excluded.

High-throughput PCR protocols have been improved significantly since that work and more recent studies have refuted the need for PCR in triplicate [14–16]. Two of these studies represented small datasets focusing on stool and soil samples [14, 16]. Marotz *et al.* studied 96 samples from diverse datasets, but once again these were largely represented by stool or environmental samples and lacked controls [15]. In our study, we find no significant difference in alpha and beta diversity or microbial composition between sample replicates from different PCR pooling strategies. It is plausible that additional manual handling from PCR in triplicate or duplicate compared to a single reaction may be a source of contamination but we did not observe this – though in our experiment, all samples were prepared in parallel and therefore had the same exposure time. Importantly, this work validates findings from prior studies. We utilized healthy human nasal samples and a diverse, serially diluted, mock community, obtaining consistent findings across the contrasting sample types. This, in addition to the reproducibility of results from multiple technical replicates, from the replicate study (experiment 2), and the use of appropriate controls to examine for the introduction of external bias in our study, provide confidence that multiple PCR amplifications are unnecessary to reduce PCR drift for 16S rRNA gene sequencing.

Performing PCR amplification in triplicate or duplicate can also help to generate a high enough PCR yield without running additional cycles, which has been shown to introduce bias [11–13]. Although we have not specifically investigated PCR cycle variation by pooling strategy replicate, we observe similarly high yields of high-quality reads from PCR amplifications in this study regardless of the PCR pooling strategy. This suggests that the PCR yield from a single reaction is sufficient for nasal samples, indicating that there may be potential to reduce the number of PCR cycles in our PCR protocol (and therefore reduce associated bias). Further work that formally examines the potential for bias from a single PCR reaction and varied PCR cycles from low-biomass samples would be useful.

Mastermix preparation varies across studies and is less studied as a source of PCR bias. In order to establish a streamlined, reproducible and standardized high-throughput 16S rRNA gene sequencing protocol, we aimed to evaluate the effect of switching to a commercially available premixed mastermix. We found no demonstrable difference in PCR efficiency (high-quality read counts), alpha and beta diversity, and microbial composition, between sample replicates from different mastermix preparations. Previous studies have reported increased contamination of premixed mastermix solutions; we found that the premixed and manually prepared mastermix solutions can both be subject to contamination (although each contained different contaminants) and including controls is essential to identify and correct for possible sources of contamination. In fact, contamination of low-biomass samples is likely to be a greater source of uncertainty, and we demonstrate through the inclusion of multiple negative controls, the mock community as a positive control and dilution series, that these contaminants can be identified and therefore accounted for. The contaminants were observed to be predominantly in low abundance in in our mock microbial community and the majority of contaminants were removed by excluding the lowest abundance species. If seen in high abundances in the controls (e.g. *M. lacunata *), the contaminants were not seen at similar levels in the samples or in the replicate experiment, suggesting a lack of gross contamination. In this instance, through careful examination of the possible sources of contamination, batch effects were identified across isolated rows in the two experiments that related to contamination of the primer stock. The use of dual-index primers (8×12) may offer some advantages over unique indexing, as contaminated primers can be identified more easily through row or column effects. This is especially useful when considering low-biomass samples, where contamination is a greater issue. Additional contaminants, such as *S. bongori *, could be identified through evaluation of laboratory practices. Furthermore, we saw greater variation in the expected species of ‘very low’ abundance (i.e. <0.1%) in our mock community *between* experiments rather than in replicates within the same experiment. This may be explained by the stochasticity of the PCR process; the ‘law of small numbers’ where variation is higher in sampling from small populations [33]. As a result, we advise particular caution when interpreting associations of phenotypes with low-abundance species that may be either contaminants or over/under-represented ‘true’ species, due to the variability between experiments, emphasizing the need for careful validation of true lower abundance taxa through the analysis of technical replicates.

Cost and turnaround time reduction are imperative to 16S rRNA gene sequencing being scaled for research and being adopted more routinely in clinical settings to evaluate culture-negative samples [34]. The practice of setting up multiple PCR amplifications per sample results in greater use of PCR plates and thermocyclers, a potential loss of reaction volume to surfaces with increased pipetting, and, although not demonstrated here, a theoretical increased risk of contamination with greater need for handling. Replacing this with a single larger-volume PCR amplification would reduce operator time, and in turn reduce costs, for both manual and automated PCR protocols by reducing the overall number of reactions prepared per sample and removing the need to pool, in addition to minimizing the risks and inefficiencies highlighted above. The use of a premixed mastermix provides a further reduction in the manual handling steps. These process improvements should result in a streamlined, more easily scalable 16S rRNA gene sequencing protocol (see the protocol in Supplementary Methods and Results and protocols.io) [32].

This study has some limitations. Although our findings have been validated on nasal (and therefore low-biomass) samples and a serially diluted diverse mock community that provides an *in vitro* representation of varying biomass, they should ideally be confirmed in samples from other niches. As discussed above, we do not evaluate the reduction in PCR amplifications in conjunction with varied PCR cycles to assess the impact on PCR yield. Further, this study does not formally investigate the replicability of each pooling strategy from the same sample and within the same experiment (i.e. three replicates of a single PCR amplification, three replicates of PCR amplification in duplication and three replicates of PCR amplification in triplicate). This comparison is partially visible in Fig. 3, when comparing single PCR amplification and triplicate pooled PCR products of mock community samples that utilize different PCR mastermixes – there is consistency of composition between replicates of the same sample by the PCR pooling method but specific examination of this possible phenomenon would be of interest. Different premixed mastermixes should preferably be validated in the same manner as this study prior to incorporation into a high-throughput 16S rRNA gene amplification protocol. Finally, although we extensively investigated the requirement to conduct PCR in triplicate, duplicate or as a single reaction, and the use of different mastermix solutions, we did have a small number of failed libraries in the second experiment and insufficient DNA from CARRIAGE samples to repeat experiment 1 on these samples specifically. However, we could validate our findings on a number of technical replicates and for the samples we tested in experiment 2 we demonstrated consistency, providing confidence in our conclusions.

## Conclusions

16S rRNA gene amplicon analysis remains a vital tool to understand the microbiome of various niches and the optimizations outlined here provide a streamlined workflow that allows for better powered studies in larger populations. This study provides vital insights to facilitate the realization of a more efficient 16S rRNA gene PCR protocol [32] by demonstrating no requirement for PCR to be conducted in triplicate or duplicate, or the manual preparation of PCR mastermix. Moreover, we strongly encourage the use of sufficient controls to account for contaminants, which are an important source of bias.

## Supplementary Data

Supplementary material 1
