# Supplementary material for: Optimization of high-throughput 16S rRNA gene amplicon sequencing: an assessment of PCR pooling, mastermix use and contamination
Source: Microb Genom. 2023 Oct 16;9(10):001115. doi: 10.1099/mgen.0.001115 (PMC10634443; doi:10.1099/mgen.0.001115)
Supplement: Supplementary material 1 [file mgen-9-1115-s001.pdf]

# Supplementary Materials

## Supplementary Figures

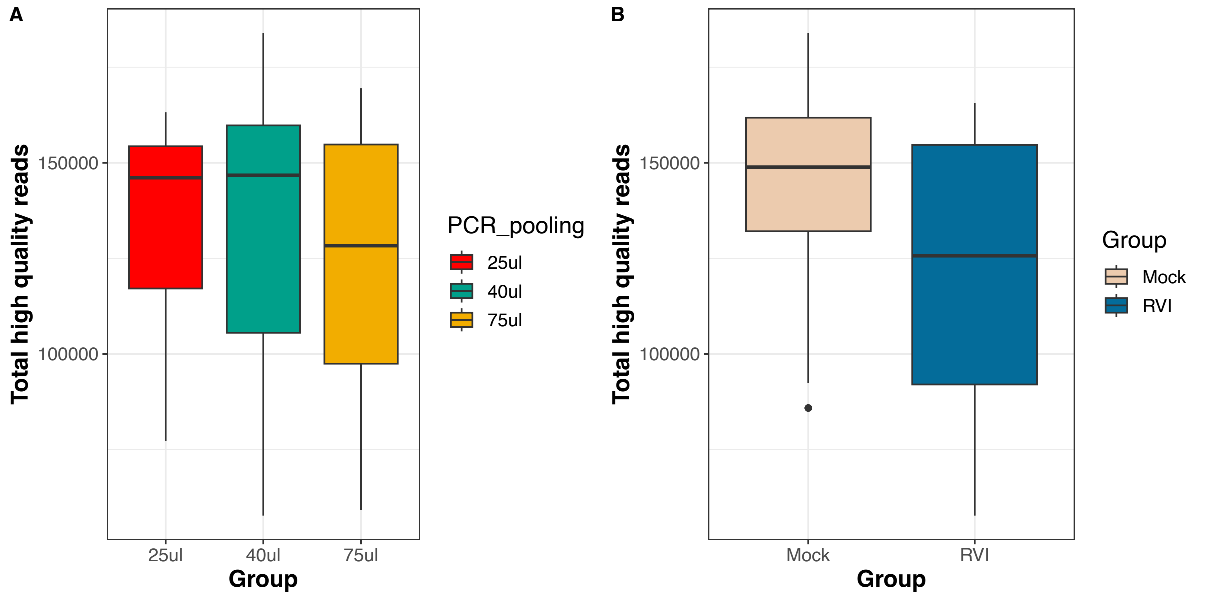


**Supplementary Figure 1:** Boxplot of high-quality reads post mothur qc. **(A)** Comparing PCR reaction replicates conducted in triplicate at 25µl (red), in duplicate at 40µl (green), and as a single 75µl reaction (yellow), from healthy nasal swabs. **(B)** Comparing healthy nasal swabs (RVI, Respiratory Virus and Microbiome Initiative) (blue) with mock community experiments (beige). Data presented are from Experiment 1.

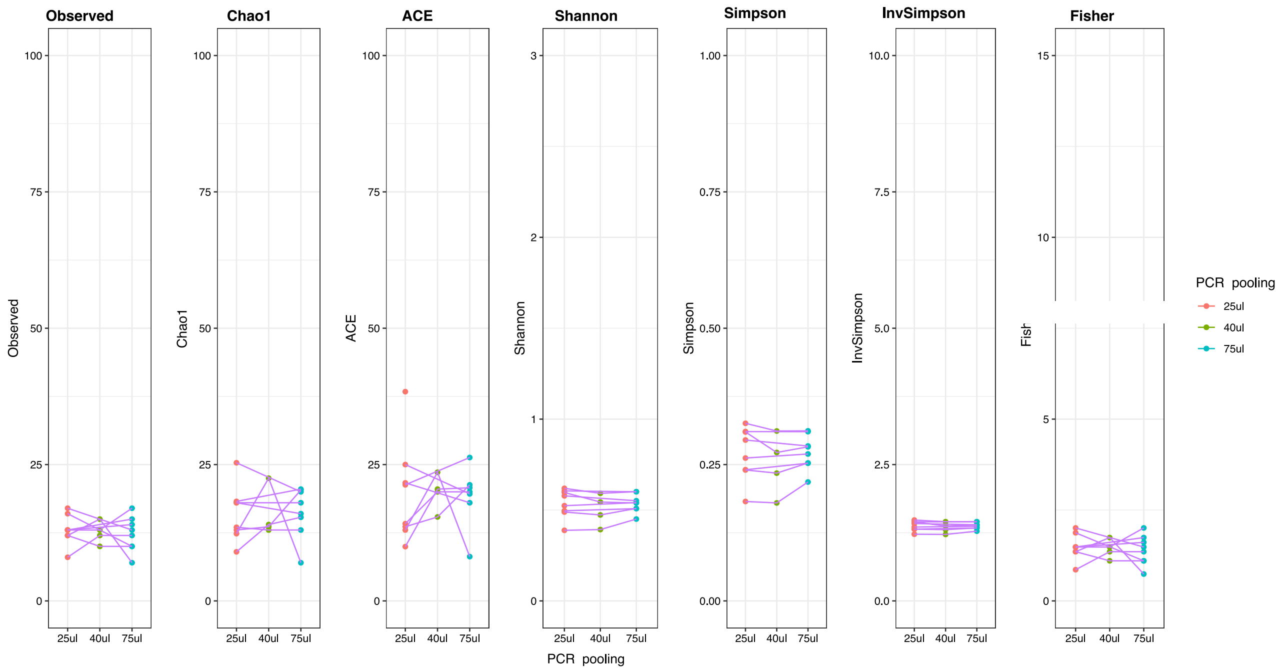

**Supplementary Figure 2:** Alpha diversity by multiple indices of mock community replicates. Plot comparing PCR reaction conducted in triplicate (25µl), duplicate (40µl), and as a single reaction (75µl). Replicates by type of mastermix preparation - manual (green) and premixed (blue) are linked by a red-line. Alpha diversity is calculated after rarefication of high-quality reads. Note, y-axis is adjusted to reflect alpha indices variation seen amongst nasal samples (Supplementary Figures 4, 5, and 15). Data presented are from Experiment 1.

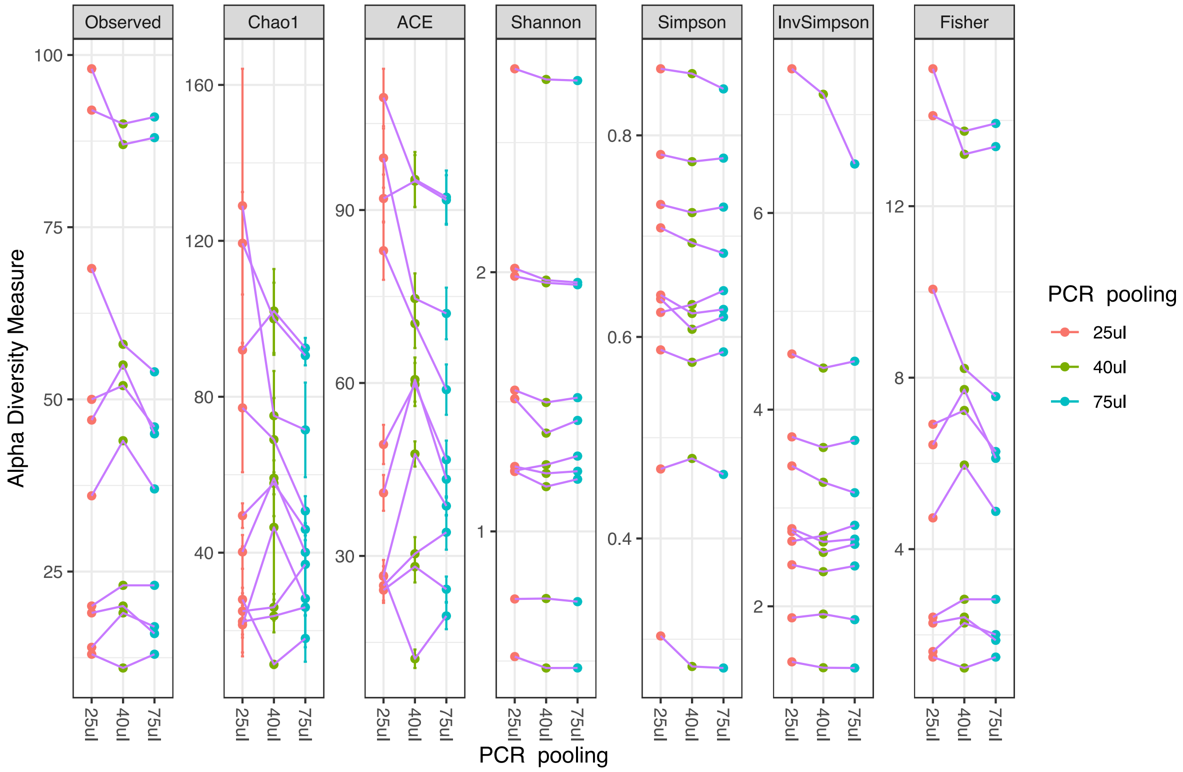


**Supplementary Figure 3:** Alpha diversity by multiple indices post mothur qc of healthy nasal swabs obtained from Healthy Volunteers at the Wellcome Sanger Institute. Plot comparing PCR reaction replicates conducted in triplicate at 25µl (red), duplicate at 40µl (green), and as a single 75µl reaction (blue), linked by purple-line. Alpha diversity is calculated after rarefication of high-quality reads. Data presented are from Experiment 1.

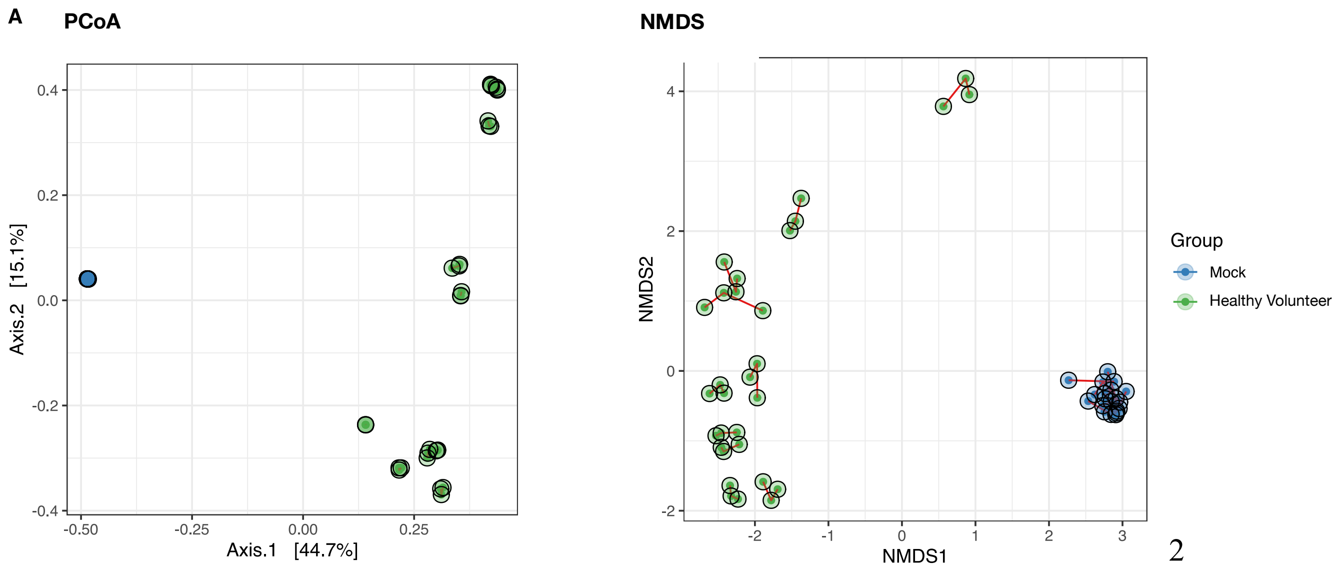

**Supplementary Figure 4:** Ordination plots of Bray-Curtis dissimilarity indices between replicate samples from different PCR pools. Principle Component Analysis (PCoA) (**A**) and Non-metric multidimensional scaling (NMDS) (**B**) of Bray-Curtis dissimilarity indices. Nasal samples obtained from healthy participants at the Wellcome Sanger Institute (green) and mock community isolates (blue) are represented. Replicates from different PCR pools are linked by a red-line. Replicates by various PCR pooling strategies cluster (no significant difference by PERMANOVA analysis, p=0.99), whereas the mock community samples are clearly distinct from nasal samples (significant difference by PERMANOVA analysis, p=0.001). In the PCoA plot, replicates cluster very closely, such that the red 'group' line is not visible. Data presented are from Experiment 1.

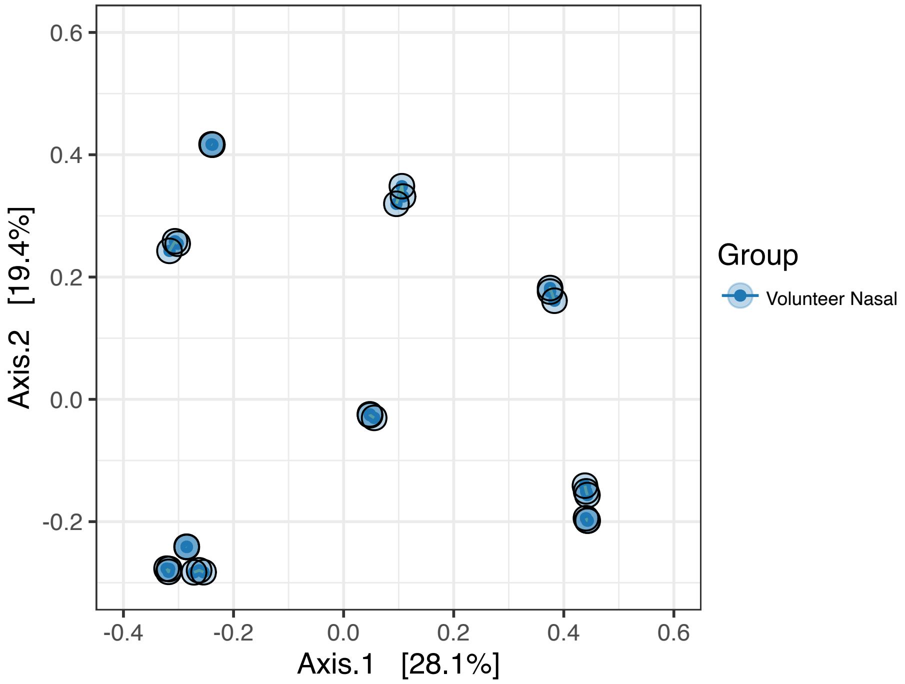


**Supplementary Figure 5:** Principle Component Analysis of Bray-Curtis dissimilarity indices between replicate samples from PCR pooling strategies (linked by green line) from nasal samples of healthy volunteers via the Wellcome Sanger Institute (blue). Replicates from libraries with different PCR pooling strategies are nearly indistinguishable. High-quality reads from the Operational Taxonomic Unit Matrix are rarefied and then converted to percentage abundance for each sample. Data presented are from Experiment 1.

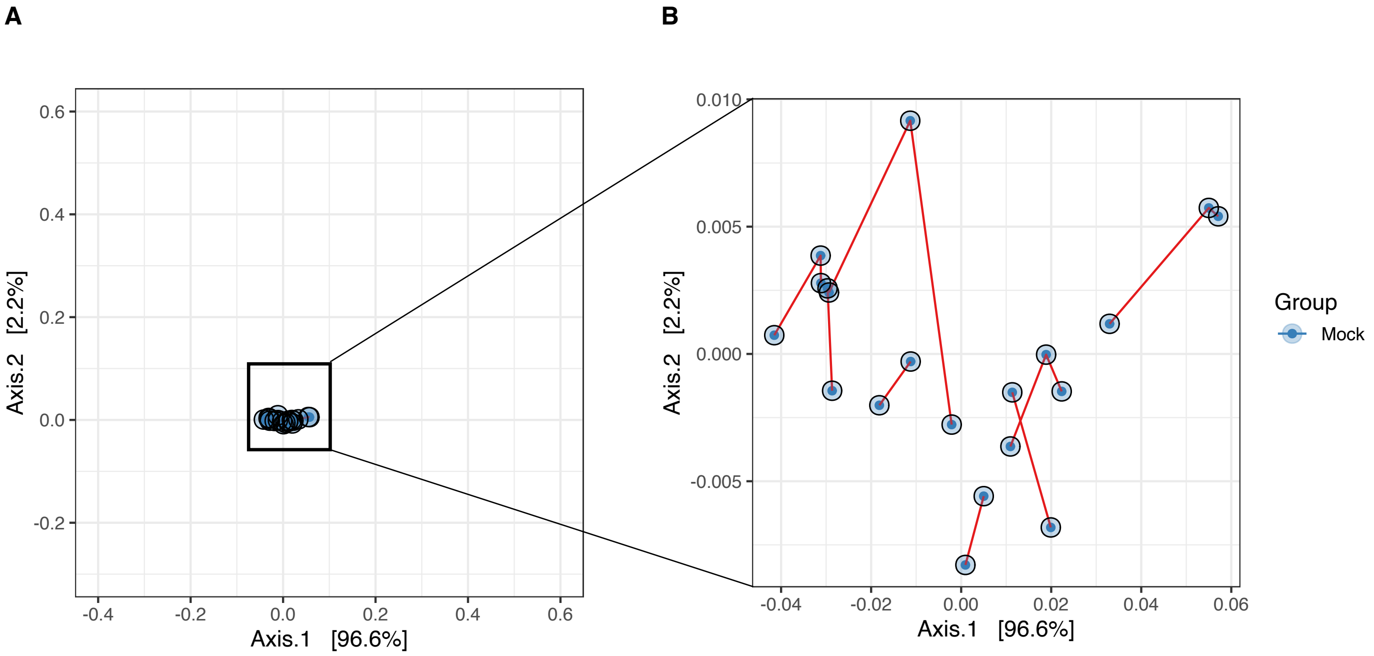


**Supplementary Figure 6:** Principle Component Analysis of Bray-Curtis dissimilarity indices between replicate samples from PCR pooling strategies (linked by red line) from mock community serially diluted preparations. **(A)** Axis of PCoA is consistent with the PCoA plot in Supplementary Figure 8 and the mock community samples are observed to closely cluster. **(B)** Axis of PCoA is exaggerated to allow closer examination of the mock community cluster. High-quality reads from the Operational Taxonomic Unit Matrix in **A** and **B** are rarefied and then converted to percentage abundance in each sample. Data presented are from Experiment 1.

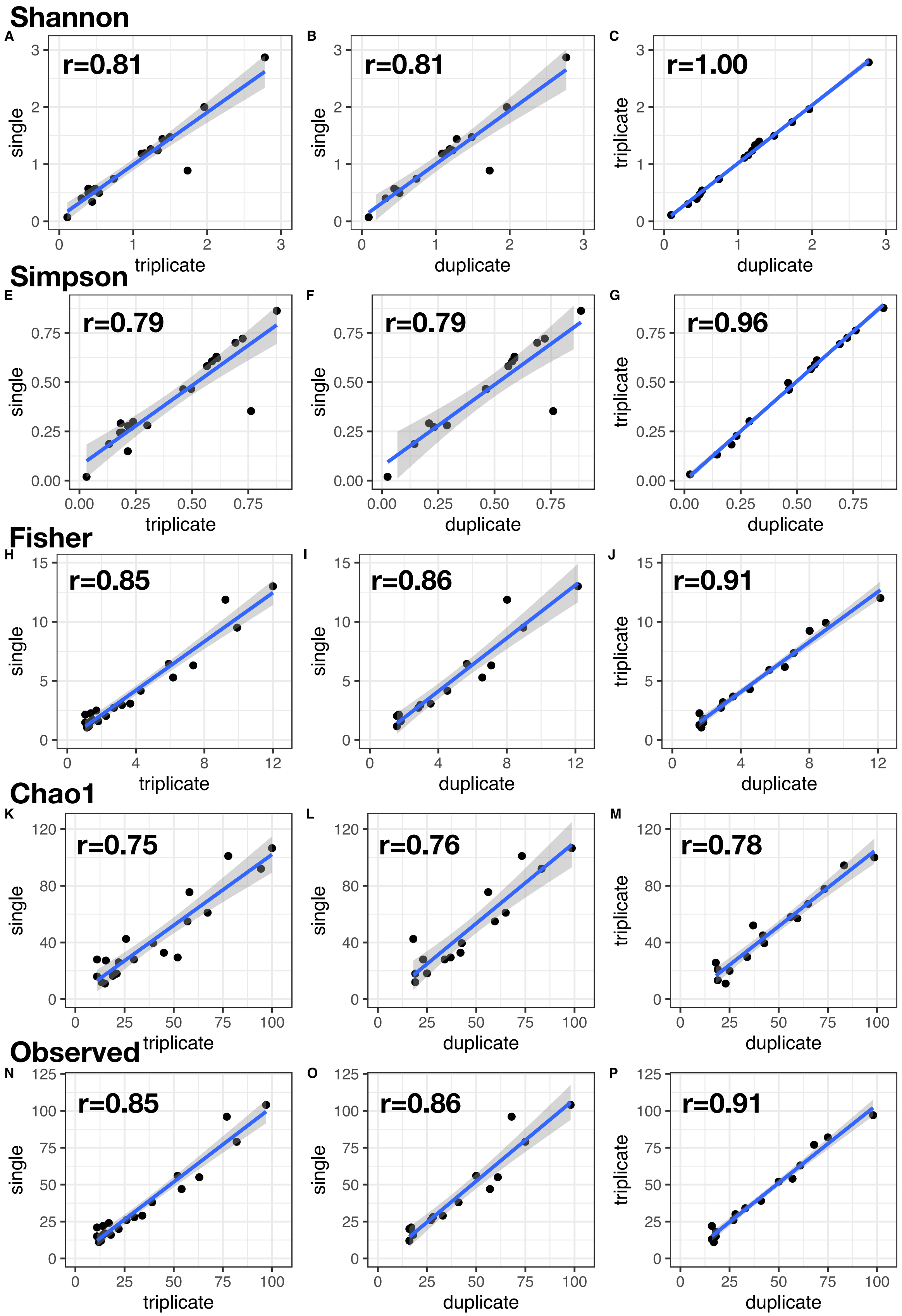


**Supplementary Figure 7:** Correlation of alpha diversity indices by PCR pooling strategy, after rarefication of high-quality sample reads with controls removed. Replicates are from healthy volunteer nasal samples from the Wellcome Sanger Institute and the serially diluted mock microbial community. Alpha indices represented include Shannon, Simpson, Fisher, Chao1,

and observed richness (**A-P**). Pairwise Kendall's rank correlation coefficient (**r**) is presented in the top-left of each plot. A strong correlation between PCR pools is observed by all alpha indices. A linear regression model is fitted to the observed values. Data presented are from Experiment 2.

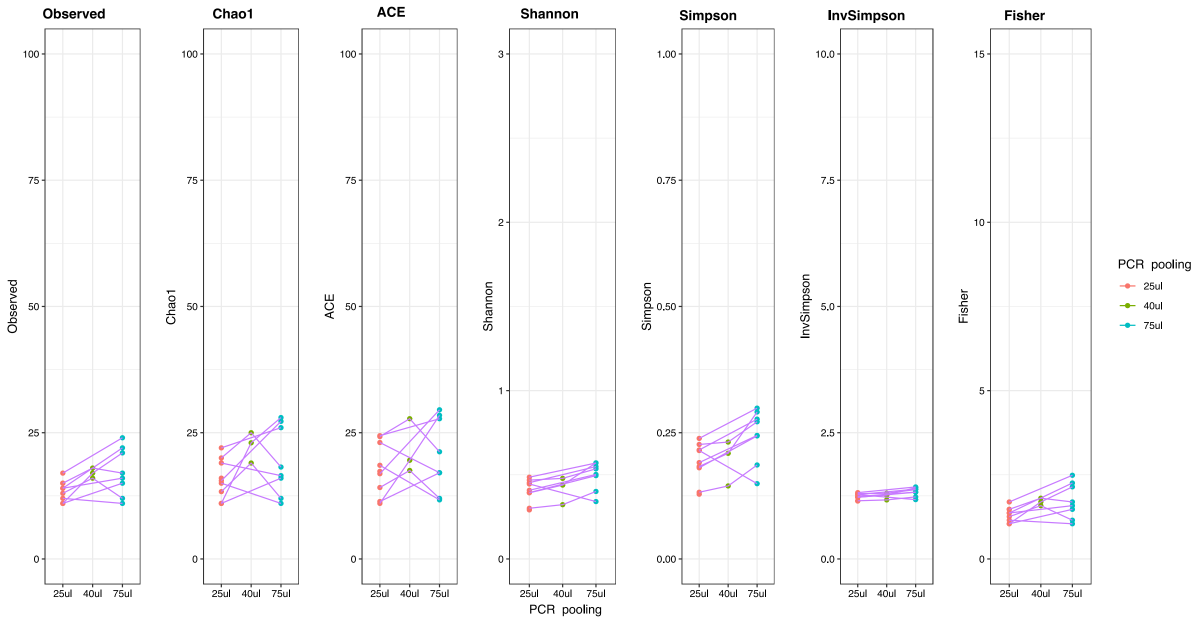


**Supplementary Figure 8** Alpha diversity by multiple indices of mock community replicates. Plot comparing PCR reaction conducted in triplicate (25µl), duplicate (40µl), and as a single reaction (75µl). Replicates by type of mastermix preparation - manual mix (red) and premixed (green) are linked by a blue-line. Alpha diversity is calculated after rarefication of high-quality reads. Note, y-axis is adjusted to reflect alpha indices variation seen amongst nasal samples (Supplementary Figures 4, 5, and 15). Data presented are from Experiment 2.

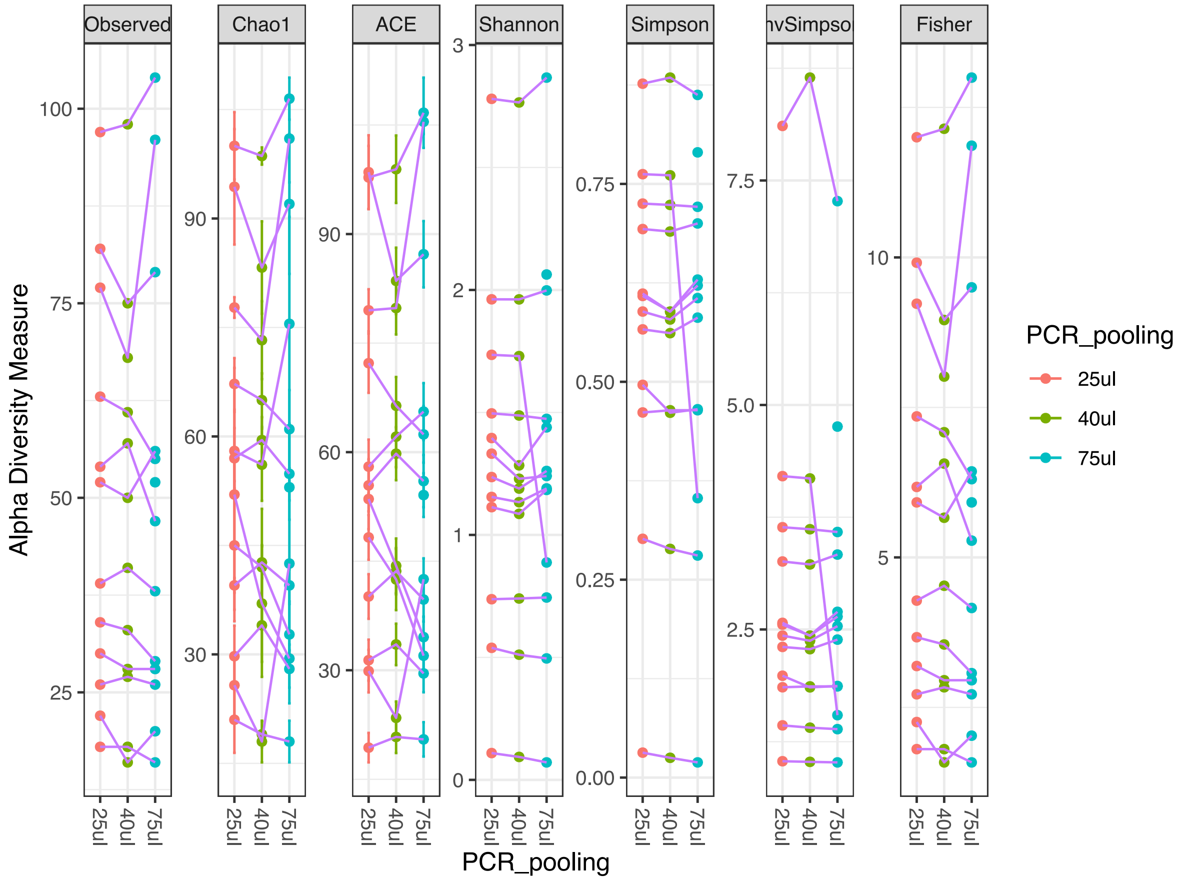


**Supplementary Figure 9:** Alpha diversity by multiple indices post mothur qc of healthy nasal swabs obtained from the Healthy Volunteers at the Wellcome Sanger Institute. Plot comparing PCR reaction replicates conducted in triplicate at 25µl (red), duplicate at 40µl (green), and as a single 75µl reaction (blue), replicates linked by purple-line. Alpha diversity is calculated after rarefication of high-quality reads. Data presented are from Experiment 2.

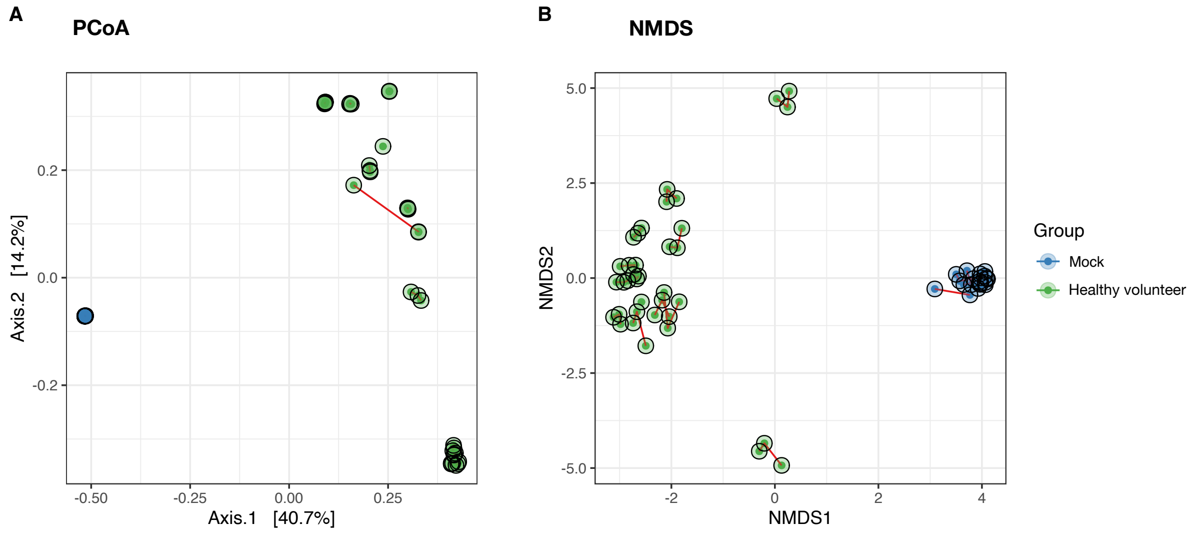

**Supplementary Figure 10:** Ordination plots of Bray-Curtis dissimilarity indices between replicate samples from different PCR pools. Principle Component Analysis (PCoA) (**A**) and Non-metric multidimensional scaling (NMDS) (**B**) of Bray-Curtis dissimilarity indices. Nasal samples from healthy volunteer obtained via the Wellcome Sanger Intitute(green) and mock community isolates (blue) are represented. Replicates from different PCR pools are linked by a red-line. Replicates from different PCR pooling strategies cluster (no significant difference by PERMANOVA analysis, p=0.94), whereas the mock community samples are clearly distinct from nasal samples (significant difference by PERMANOVA analysis, p<0.001). In the PCoA plot, replicates cluster very close, such that the red line connecting them is not visible. Data presented are from Experiment 2.

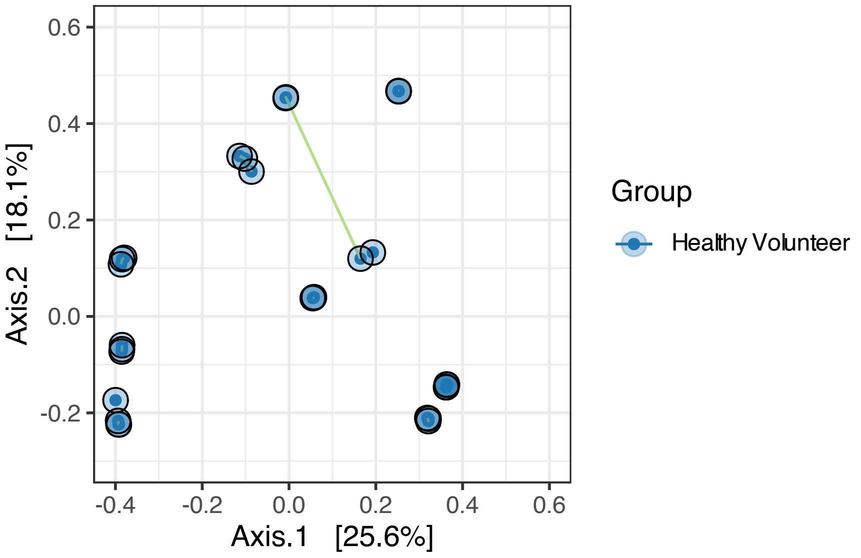


**Supplementary Figure 11:** Principle Component Analysis of Bray-Curtis dissimilarity indices between replicate samples from PCR pooling strategies (linked by green line) from nasal samples of healthy volunteers obtained via the Wellcome Sanger Institute (blue). Replicates from libraries with different PCR pooling strategies are nearly indistinguishable. High-quality reads from the Operational Taxonomic Unit Matrix are rarefied and then converted to percentage abundance in each sample. Data presented are from Experiment 2.

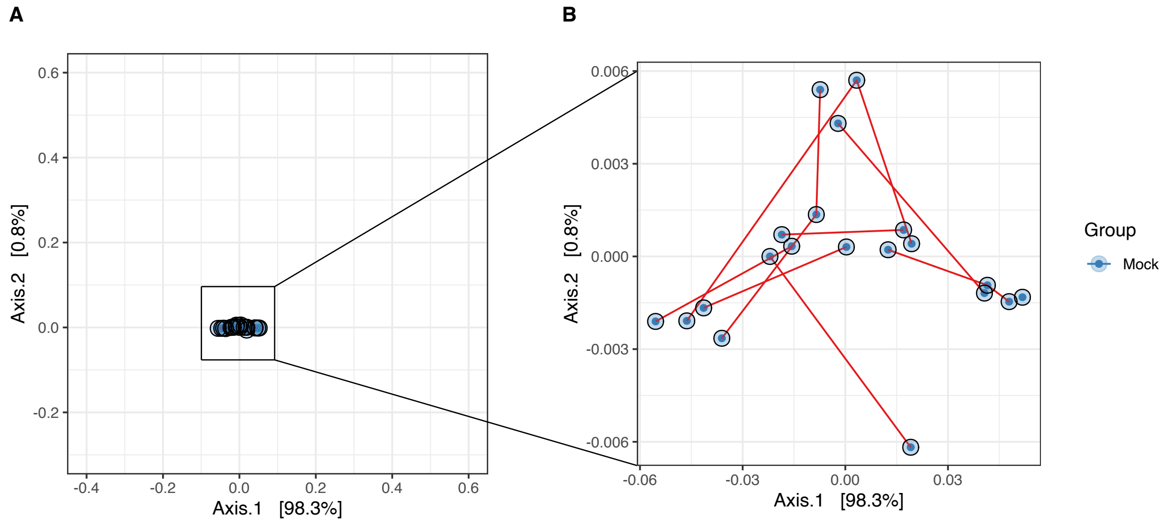

**Supplementary Figure 12:** Principle Component Analysis of Bray-Curtis dissimilarity indices between replicate samples from PCR pooling strategies (linked by red line) from mock community serially diluted preparations. **(A)** Axis of PCoA is consistent with the PCoA plot in Supplementary Figure 11and the mock community samples are observed to closely cluster. **(B)** Axis of PCoA is exaggerated to allow closer examination of the mock community cluster. High-quality reads from the Operational Taxonomic Unit Matrix in **A** and **B** are rarefied and then converted to percentage abundance in each sample. Data presented are from Experiment 2.

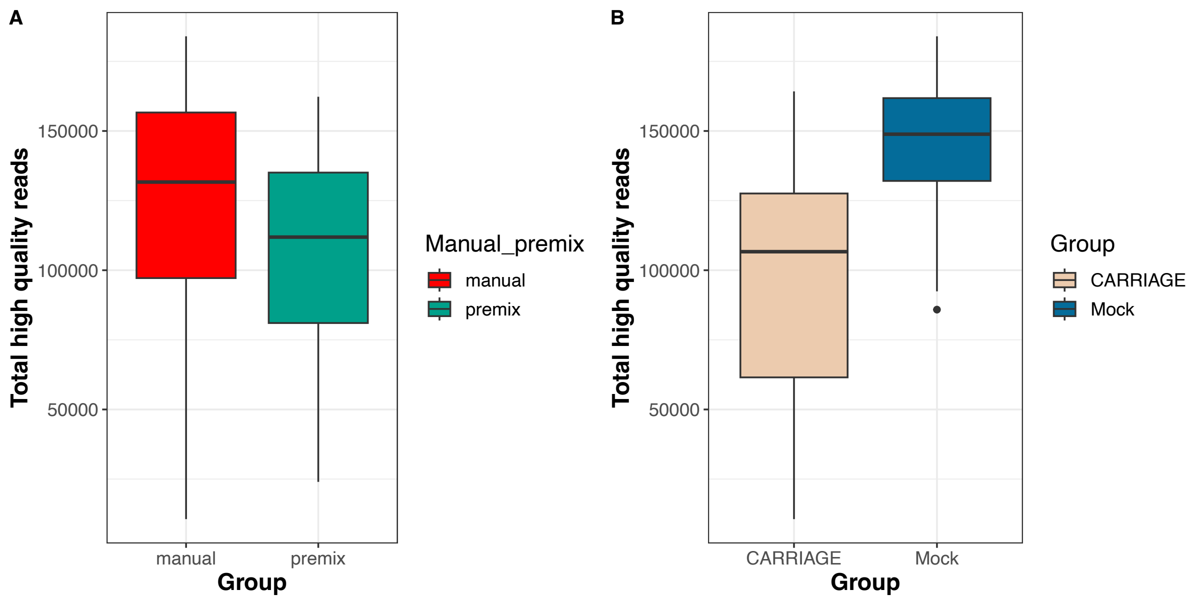


**Supplementary Figure 13:** Boxplot of high-quality reads post mothur qc. **(A)** Comparing manual (red) and premixed (green) mastermix replicates from healthy nasal swabs (CARRIAGE study). **(B)** Comparing healthy nasal swabs (CARRIAGE) (beige) with mock community experiments (blue). Data presented are from Experiment 1.

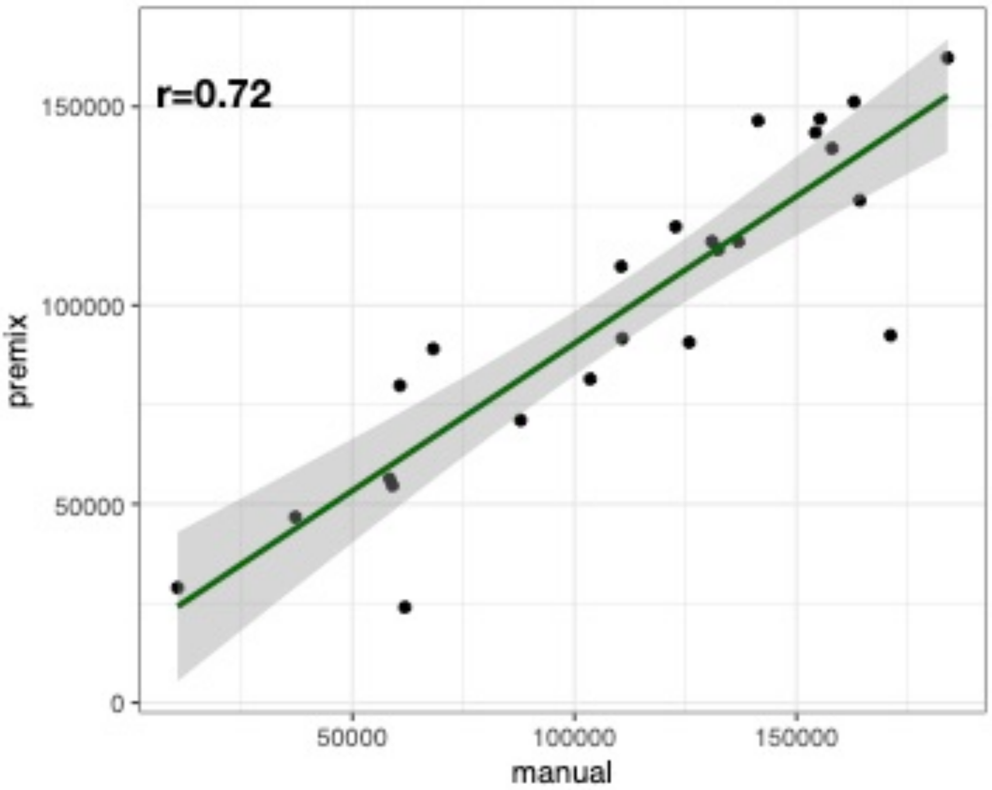


**Supplementary Figure 14.** Correlation of high-quality reads obtained from samples by comparing mastermix preparations (premixed vs manual). Replicates are from CARRIAGE nasal samples from the Wellcome Sanger Institute and the serially diluted mock microbial community. Pairwise Kendall's rank correlation coefficient (r) is presented in the top-left of the plot. A linear regression model is fitted to the observed values. Data presented are from Experiment 1.

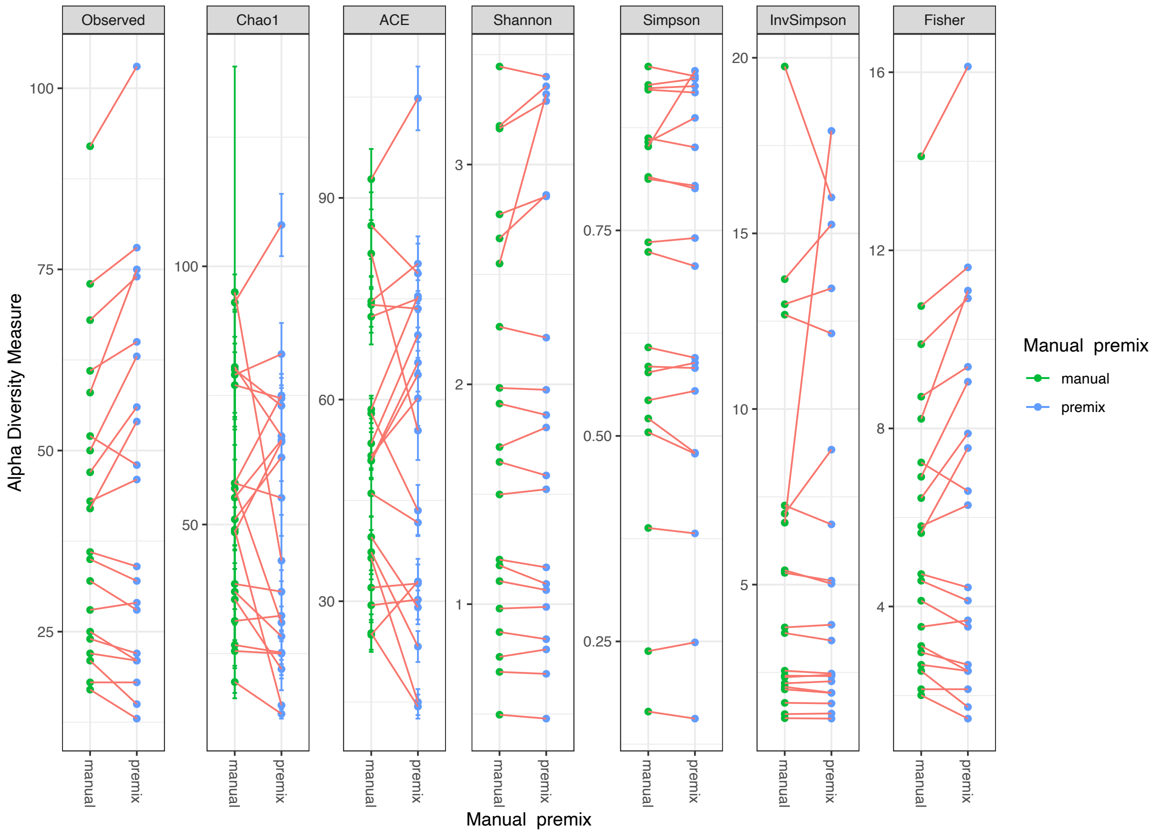


**Supplementary Figure 15:** Alpha diversity by multiple indices post mothur qc of healthy nasal swabs from the CARRIAGE study, comparing manual (green) and premixed (blue) mastermix replicates (linked by red-line). Alpha diversity is calculated after rarefication of high-quality reads. Data presented are from Experiment 1.

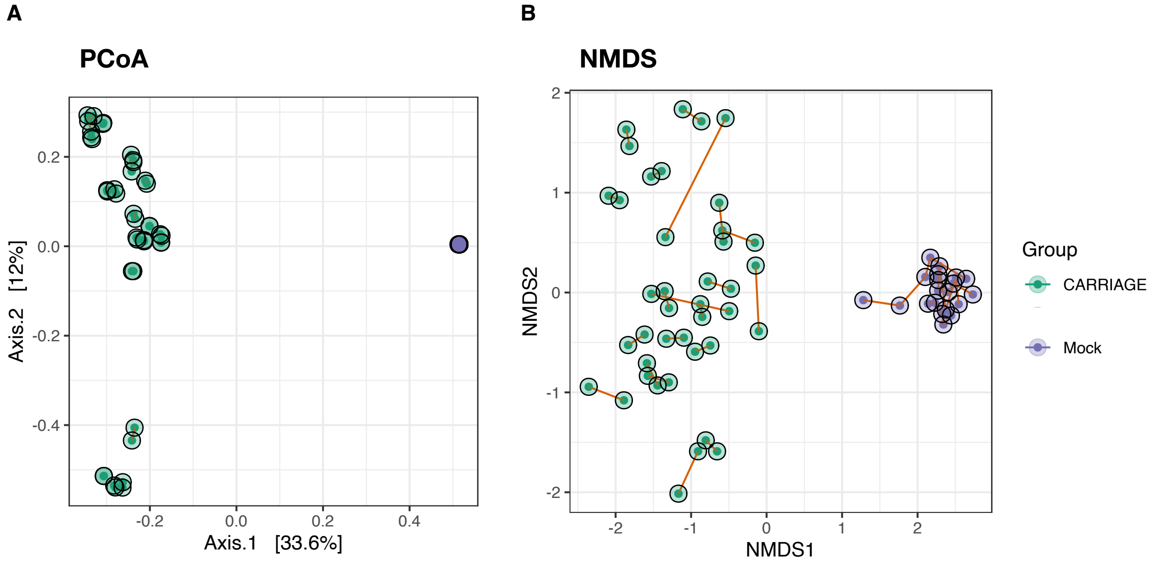


**Supplementary Figure 16:** Ordination plots of Bray-Curtis dissimilarity indices between replicate samples from different mastermix preparations. Principle Component Analysis (PCoA) (**A**) and Non-metric multidimensional scaling (NMDS) (**B**) of Bray-Curtis dissimilarity indices. Nasal samples from healthy participants (CARRIAGE) (green) and mock community

isolates (purple) are represented. Replicates from different mastermix preparations are linked by a red-line. Replicates from individuals with different mastermix preparations cluster (no significant difference by PERMANOVA analysis, p=1), whereas the mock community samples are clearly distinct from nasal samples (significant difference by PERMANOVA analysis, p=0.001). In the PCoA plot, replicates cluster very close, such that the red line connecting them is not visible. Data presented are from Experiment 1.

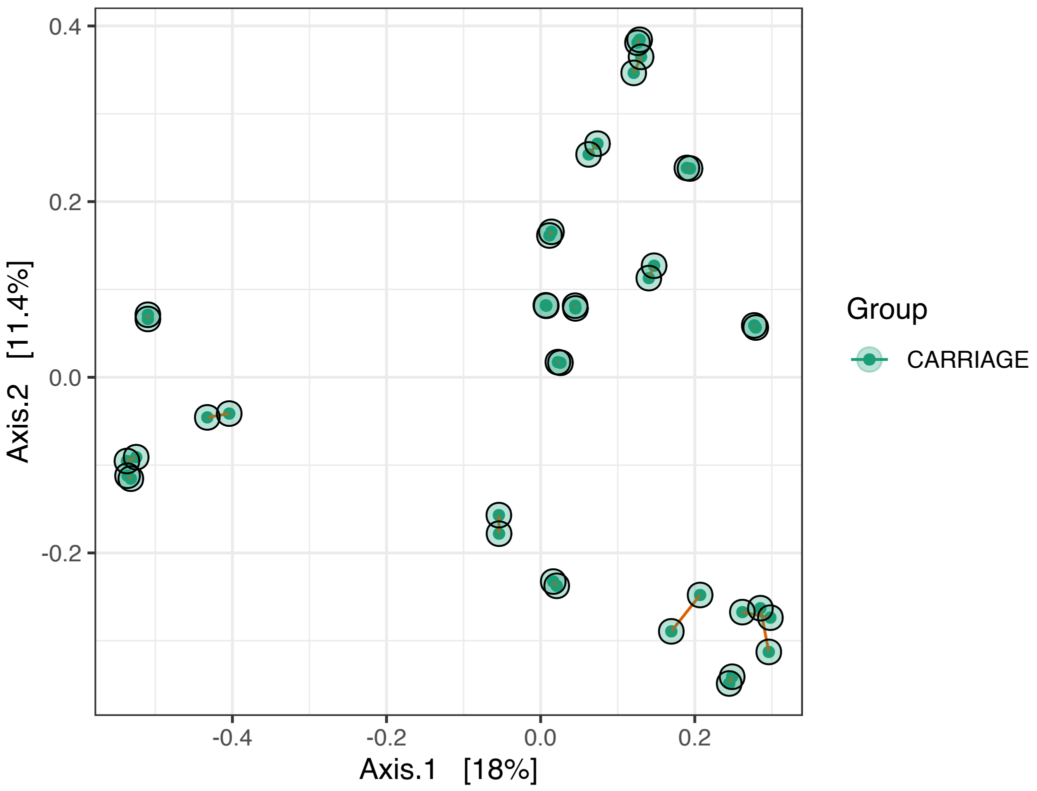


**Supplementary Figure 17:** Principle Component Analysis of Bray-Curtis dissimilarity indices between replicate samples with PCR using manually prepared or premixed mastermix (linked by red line) from healthy nasal samples of CARRIAGE participants (green). Replicates from libraries with different mastermixes used are nearly indistinguishable. High-quality reads from the Operational Taxonomic Unit Matrix are rarefied and then converted to percentage abundance in each sample. Data presented are from Experiment 1.

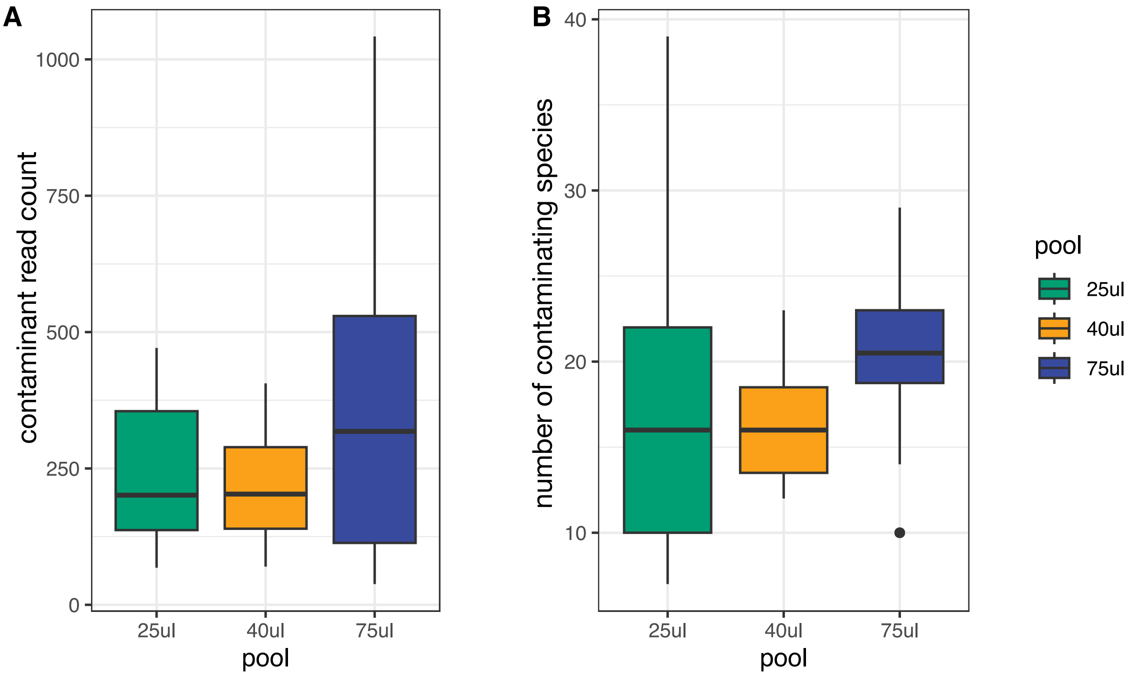


**Supplementary Figure 18: (A)** Boxplot of high-quality contaminant reads in the mock microbial community samples by pooling strategy - in triplicate at 25µl (red), in duplicate at 40µl (green), and as a single 75µl reaction (yellow). **(B)** Boxplot of number of contaminating species in the mock microbial community samples by pooling strategy - in triplicate at 25µl (red), in duplicate at 40µl (green), and as a single 75µl reaction (yellow).

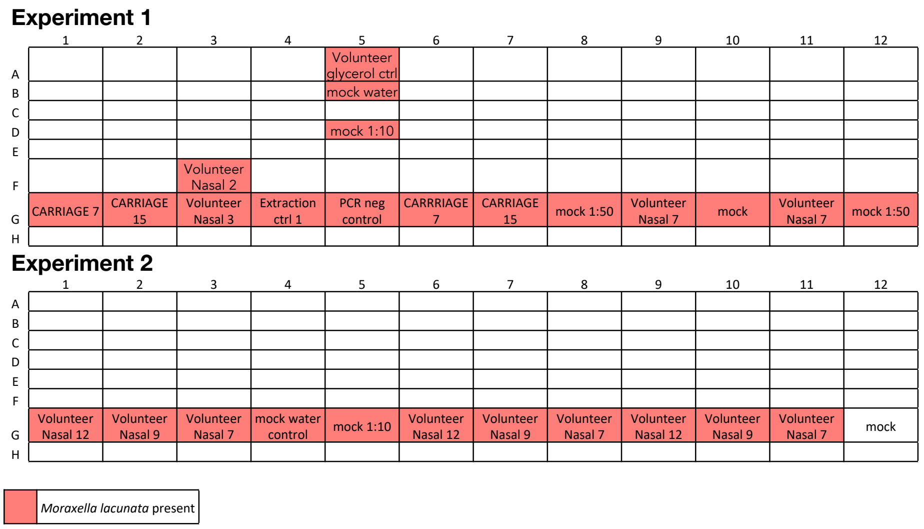


**Supplementary Figure 19**: Distribution of *Moraxella lacunata* on plate map of Experiment 1 and Experiment 2. Contamination of *Moraxella lacunata* is seen in Row G on the plate maps of both experiments demonstrating a batch effect related to primer stock contamination. 1 or 2 reads mapping to *Moraxella lacunata* are seen in samples outside of Row G in plate 1 and none in plate 2.

**Supplementary Tables**

**Supplementary Table 1:** Differences in alpha diversity between PCR pooling strategies using Kruskall-Wallis tests. No significant difference is

| **EXPERIMENT 1** | | |
|---|---|---|
| ALPHA INDEX | **chi-squared** | **p-value** |
| SHANNON | **0.15** | **0.93** |
| SIMPSON | **0.23** | **0.89** |
| FISHER | **0.68** | **0.71** |
| CHAO1 | **0.56** | **0.76** |
| OBSERVED | **0.68** | **0.71** |
| **EXPERIMENT 2** | | |
| ALPHA INDEX | | |
| SHANNON | **0.15** | **0.93** |
| SIMPSON | **0.23** | **0.89** |
| FISHER | **0.68** | **0.71** |
| CHAO1 | **0.56** | **0.76** |
| OBSERVED | **0.68** | **0.71** |

seen by Shannon, Simpson, Fisher, Chao1, and Observed indices between PCR pool strategies (chi-squared and p-values presented to 2 d.p.). Alpha diversity is calculated after rarefication of high-quality reads.

**Supplementary Table 2:** Relative abundance of mock community by PCR pool strategy compared to manufacturer reported. Data presented are from Experiment 1.

| Sample | Listeria monocytogenes | Pseudomonas aeruginosa | Bacillus subtilis | Salmonella enterica | Escherichia coli | Lactobacillus fermentum | Staphylococcus aureus | Enterococcus faecalis |
|---|---|---|---|---|---|---|---|---|
| **Mock** | 80.5546932 | 16.393107 | 2.28931499 | 0.39498279 | 0.33685824 | 0.02708076 | 0.00066051 | 0.00330253 |
| **Mock_40ul** | 81.8466226 | 15.2278354 | 2.27046438 | 0.33297968 | 0.29489377 | 0.02339563 | 0 | 0.00380859 |
| **Mock_75ul** | 81.4774762 | 15.5158048 | 2.31354265 | 0.37111233 | 0.2954716 | 0.02363773 | 0.00118189 | 0.00177283 |
| **Mock_premix** | 81.6446133 | 14.9945722 | 2.53124633 | 0.44595705 | 0.36307358 | 0.01466964 | 0.00513437 | 0.00073348 |
| **Mock_premix_75ul** | 82.0907493 | 14.6272248 | 2.52691323 | 0.38249175 | 0.33313797 | 0.03022919 | 0.00616922 | 0.00308461 |
| **Mock 1:10** | 82.1439959 | 14.5661062 | 2.41906341 | 0.45490296 | 0.37837723 | 0.03684572 | 0 | 0.00070857 |
| **Mock 1:10_40ul** | 84.2815218 | 12.7867764 | 2.22404961 | 0.39909167 | 0.27612013 | 0.03168592 | 0 | 0.00075443 |
| **Mock 1:10_75ul** | 83.5050724 | 14.0669732 | 1.74465581 | 0.34913066 | 0.30424244 | 0.02793045 | 0.00099752 | 0.00099752 |
| **Mock 1:10_premix** | 82.6928655 | 13.9633643 | 2.57401885 | 0.39937825 | 0.34359916 | 0.02231163 | 0.00297488 | 0.00148744 |
| **Mock 1:10_premix_75ul** | 83.4823472 | 13.2637101 | 2.5231034 | 0.40435225 | 0.32033987 | 0 | 0.00478119 | 0.00136605 |
| **Mock 1:50** | 86.6069345 | 10.8782962 | 1.94057898 | 0.2867881 | 0.2530122 | 0.02947715 | 0.00184232 | 0.00307054 |
| **Mock 1:50_40ul** | 86.9211273 | 10.4881271 | 2.04466018 | 0.30464489 | 0.21426269 | 0.02085743 | 0.00316022 | 0.00316022 |
| **Mock 1:50_75ul** | 85.4585188 | 11.7674007 | 2.12590772 | 0.30396642 | 0.29715658 | 0.03962088 | 0.00185723 | 0.00557169 |
| **Mock 1:50_premix** | 85.1549517 | 11.8845018 | 2.31812027 | 0.33467717 | 0.27312735 | 0.02692805 | 0.00769373 | 0 |
| **Mock 1:50_premix_75ul** | 84.7011715 | 12.2483288 | 2.33437024 | 0.3878483 | 0.28195115 | 0.037064 | 0.00794229 | 0.00132371 |
| **Mock1:100** | 89.8535779 | 8.09484769 | 1.68840153 | 0.155005 | 0.16525326 | 0.03843099 | 0.00448362 | 0 |
| **Mock1:100_40ul** | 89.5793153 | 8.27868757 | 1.76327571 | 0.18819184 | 0.14494278 | 0.03798904 | 0.00759781 | 0 |
| **Mock1:100_75ul** | 87.9312632 | 9.71117753 | 1.90259168 | 0.26299155 | 0.19197603 | 0 | 0 | 0 |
| **Mock1:100_premix** | 86.2931075 | 10.8976154 | 2.3099538 | 0.28932755 | 0.20532923 | 0.00116664 | 0 | 0.00349993 |
| **Mock1:100_premix_75ul** | 86.4103008 | 11.0253189 | 1.96551948 | 0.29022547 | 0.27506443 | 0.03140499 | 0 | 0.00216586 |
| **Actual** | **0.959** | **0.028** | **0.012** | **0.0007** | **0.00069** | 0.00012 | **0.000001** | **0.000007** |

**Supplementary Table 3:** Differences in Alpha diversity between mastermix choice using Mann-Whitney-U tests. No significant difference is seen by Shannon, Simpson, Fisher, Chao1, and Observed indices between manually prepared and premixed mastermix. Alpha diversity is calculated after rarefication of high-quality reads.

| **EXPERIMENT 1** | |
|---|---|
| ALPHA INDEX | **p-value** |
| SHANNON | **0.42** |
| SIMPSON | **0.50** |
| FISHER | **0.31** |
| CHAO1 | **0.40** |
| OBSERVED | **0.31** |
| **EXPERIMENT 2** | **p-value** |
| ALPHA INDEX | |
| SHANNON | **0.79** |
| SIMPSON | **0.85** |
| FISHER | **0.49** |
| CHAO1 | **0.56** |
| OBSERVED | **0.49** |

## Supplementary Methods and Results

*Original Protocol*

The manually prepared master mix contained 5ul 5x Q5 Buffer (NEB, USA), 0.5ul 10mM dNTPs (NEB, USA), 0.25ul Taq polymerase (NEB, USA), 14.25ul nuclease free water (ThermoFisher Scientific, USA), 1.25ul of both the forward and reverse indexed cartridge purified primers (ThermoFisher Scientific, USA) diluted to10uM in nuclease free water as before. Primer sequences are included below. Each 2.5ul aliquot of nucleic acid extraction was run with 22.5ul of master mix in triplicate. PCR was run for 32 cycles, initially at 98ºC for 2minutes, with 30 cycles of 98ºC for 30 seconds, 50ºC for 30 seconds, 72ºC for 1 minute and 30 seconds and finished with 72ºC for 5mins. Triplicate PCR products were pooled into single reactions per sample and sample pools were purified using an AMPure XP (Beckman Coulter) workflow at a ratio of 1X. Libraries were quantified using the Qubit High Sensitivity dsDNA kit (ThermoFisher) and equimolar library pools created. The equimolar pools were purified by gel electrophoresis and the Wizard SV Gel and PCR Clean Up Kit (Promega) before submission for sequencing.

*Process used in Operations to generate the data*

PCR was performed to amplify bacterial 16S ribosomal gene regions using V1V2 specific primers with attached adaptors and indexes. Manually prepared PCR reaction mastermixes were made using the Q5 High-Fidelity Polymerase Kit (New England Biolabs), according to the original protocol as described above. Pre-mixed mastermixes contained 12.5ul Q5 Hot Start High-Fidelity 2X Master Mix (New England Biolabs) and 7.5ul nuclease free water (ThermoFisher Scientific). 2.5ul nucleic acid extract was used per 25ul triplicate reaction, 4ul per duplicate 40ul reaction and 7.5ul per single 75ul reaction. 1.25ul of each forward and reverse primers (ThermoFisher Scientific) diluted to 10uM with nuclease free water were used per 25ul reaction and scaled accordingly for 40ul and 75ul reactions. Mastermix reagent volumes were scaled accordingly. PCR was run for 30 cycles, initially at 98ºC for

2minutes, with 30 cycles of 98ºC for 30 seconds, 50ºC for 30 seconds, 72ºC for 1 minute and 30 seconds and finished with 72ºC for 5mins. Triplicate and duplicate PCR products were pooled into single reactions per sample respectively and all samples were purified using an AMPure XP (Beckman Coulter) workflow at a ratio of 0.8X. Libraries were quantified using the AccuClear Ultra High Sensitivity dsDNA Quantitation kit (Biotium) and equimolar pools were subsequently created using a Biomek NX-8 liquid handler (Beckman Coulter). Samples were sequenced using the Illumina MiSeq (300bp paired-end reads, v3 Reagent Kit). Process controls included an extraction control, a negative PCR water control, an aliquot of the glycerol used for storage and an aliquot of the water used to dilute the mock community.

*Proposed optimisations*

Based on our findings, the optimised process would utilise single PCR reactions (75ul), removing the need to set up multiple reactions per sample, significantly saving time and effort. Additionally, this eliminates the requirement for subsequent pooling per sample post-PCR, removing the risk of pooling incorrect samples together, together with the increased level of sample tracking that would otherwise be necessary to support this step at scale. A pre-mixed mastermix would be used in place of a manually prepared mastermix, for speed, convenience and to reduce the risk of manual handling error.

**Full length Illumina tagged primers used in study**:

| **Name** | Sequence |
|---|---|
| V1FW_SD501 | AATGATACGGCGACCACCGAGATCTACACAAGCAGCAacactctttccctacacgacgctcttccgatctNNNNAGMGTTYGATYMTGGCTCAG |
| V1FW_SD502 | AATGATACGGCGACCACCGAGATCTACACACGCGTGAacactctttccctacacgacgctcttccgatctNNNNAGMGTTYGATYMTGGCTCAG |
| V1FW_SD503 | AATGATACGGCGACCACCGAGATCTACACCGATCTACacactctttccctacacgacgctcttccgatctNNNNAGMGTTYGATYMTGGCTCAG |
| V1FW_SD504 | AATGATACGGCGACCACCGAGATCTACACTGCGTCACacactctttccctacacgacgctcttccgatctNNNNAGMGTTYGATYMTGGCTCAG |
| V1FW_SD505 | AATGATACGGCGACCACCGAGATCTACACGTCTAGTGacactctttccctacacgacgctcttccgatctNNNNAGMGTTYGATYMTGGCTCAG |
| V1FW_SD506 | AATGATACGGCGACCACCGAGATCTACACCTAGTATGacactctttccctacacgacgctcttccgatctNNNNAGMGTTYGATYMTGGCTCAG |
| V1FW_SD507 | AATGATACGGCGACCACCGAGATCTACACGATAGCGTacactctttccctacacgacgctcttccgatctNNNNAGMGTTYGATYMTGGCTCAG |
| V1FW_SD508 | AATGATACGGCGACCACCGAGATCTACACTCTACACTacactctttccctacacgacgctcttccgatctNNNNAGMGTTYGATYMTGGCTCAG |
| V2RV_SD701 | CAAGCAGAAGACGGCATACGAGATACCTAGTAgtgactggagttcagacgtgtgctcttccgatctNNNNGCTGCCTCCCGTAGGAGT |
| V2RV_SD702 | CAAGCAGAAGACGGCATACGAGATACGTACGTgtgactggagttcagacgtgtgctcttccgatctNNNNGCTGCCTCCCGTAGGAGT |
| V2RV_SD703 | CAAGCAGAAGACGGCATACGAGATATATCGCGgtgactggagttcagacgtgtgctcttccgatctNNNNGCTGCCTCCCGTAGGAGT |
| V2RV_SD704 | CAAGCAGAAGACGGCATACGAGATCACGATAGgtgactggagttcagacgtgtgctcttccgatctNNNNGCTGCCTCCCGTAGGAGT |
| V2RV_SD705 | CAAGCAGAAGACGGCATACGAGATCGTATCGCgtgactggagttcagacgtgtgctcttccgatctNNNNGCTGCCTCCCGTAGGAGT |
| V2RV_SD706 | CAAGCAGAAGACGGCATACGAGATCTGCGACTgtgactggagttcagacgtgtgctcttccgatctNNNNGCTGCCTCCCGTAGGAGT |
| V2RV_SD707 | CAAGCAGAAGACGGCATACGAGATGCTGTAACgtgactggagttcagacgtgtgctcttccgatctNNNNGCTGCCTCCCGTAGGAGT |
| V2RV_SD708 | CAAGCAGAAGACGGCATACGAGATGGACGTTAgtgactggagttcagacgtgtgctcttccgatctNNNNGCTGCCTCCCGTAGGAGT |
| V2RV_SD709 | CAAGCAGAAGACGGCATACGAGATGGTCGTAGgtgactggagttcagacgtgtgctcttccgatctNNNNGCTGCCTCCCGTAGGAGT |
| V2RV_SD710 | CAAGCAGAAGACGGCATACGAGATTAAGTCTCgtgactggagttcagacgtgtgctcttccgatctNNNNGCTGCCTCCCGTAGGAGT |
| V2RV_SD711 | CAAGCAGAAGACGGCATACGAGATTACACAGTgtgactggagttcagacgtgtgctcttccgatctNNNNGCTGCCTCCCGTAGGAGT |
| V2RV_SD712 | CAAGCAGAAGACGGCATACGAGATTTGACGCAgtgactggagttcagacgtgtgctcttccgatctNNNNGCTGCCTCCCGTAGGAGT |

*Mis-assigned taxa by arb*

A few operational taxonomic units were evaluated with BLAST where the species was disconcordant with the taxa genus and/or not a prokaryote, as assigned by arb, or where an uncultured bacterium was seen across the mock microbial community suggesting it was either an expected mock microbial community member or a significant contaminant:

- Experiement 1
    - *Malaclemys terrapin terrapin – E. coli*, OTU 3805
    - uncultured *Enterobacteriaceae bacterium – E. coli* OTU 4256
    - uncultured *Enterobacteriaceae bacterium – E. coli* OTU 4397
    - *Coregonus lavaretus* (common whitefish): *Cutibacterium acnes* OTU 996
    - *Coregonus lavaretus* (common whitefish): uncultured *bacterium* OTU 1020
    - *Coregonus lavaretus* (common whitefish): *Cutibacterium acnes* OTU 4188
    - *Coregonus lavaretus* (common whitefish): uncultured *Propionibacterium* OTU 4327
    - *Coregonus lavaretus* (common whitefish): uncultured *Propionibacterium* OTU 2493
    - *Coregonus lavaretus* (common whitefish): uncultured *Propionibacterium* OTU 4224
    - *Coregonus lavaretus* (common whitefish): uncultured bacterium OTU 2490
    - *Amia calva* (bowfin): *Paracoccus salipaludis* OTU 2864
    - *Ambystoma mexicanum* (axolotl): *Acinetobacter lwoffii* OTU 3596
    - *Ambystoma mexicanum*: *Acinetobacter seifertii* OTU 4146
    - *Ambystoma mexicanum* (axolotl): *Acinetobacter lwoffii* OTU 3597
    - *Solanum melongena* (eggplant): *Moraxella osloensis* OTU 1489
    - unidentified marine bacterioplankton: uncultured *Enterococcaceae* bacterium OTU 4144
    - *Trichuris trichiura* (human whipworm): uncultured *Bacteroidetes* bacterium OTU 1936

- *Trichuris trichiura* (human whipworm): uncultured *Bacteroidetes* bacterium OTU 2223
- *Triticum aestivum* (bread wheat): *Pseudomonas gessardii* OTU 3978
- *Triticum aestivum (*bread wheat): *Pantoea agglomerans* OTU 4362
- *Triticum aestivum* (bread wheat): *Pantoea agglomerans* OTU 4241
- *Athetis lepigone*: *Pseudomonas putida* OTU 2630
- *Elodea nuttallii*: Uncultured *Methyloversatilis* sp. OTU 3029
- *Bryum argenteum var. argenteum*: *Pseudomonas putida* OTU 3620

- Experiment 2:
  - *Malaclemys terrapin terrapin – E. coli*, OTU 1719 - done
  - uncultured *Enterobacteriaceae bacterium – E. coli* OTU 1717
  - *Coregonus lavaretus* (common whitefish): *Cutibacterium acnes* OTU 44 - done
  - *Coregonus lavaretus* (common whitefish): uncultured *bacterium* OTU 1685 - done
  - *Coregonus lavaretus* (common whitefish): uncultured *bacterium* OTU 588 - done
  - *Coregonus lavaretus* (common whitefish): uncultured *Propionibacterium* OTU 1664 - done
  - *Coregonus lavaretus* (common whitefish): uncultured *Propionibacterium* OTU 1820 - done
  - *Coregonus lavaretus* (common whitefish): uncultured *Propionibacterium* OTU 1729 - done
  - *Coregonus lavaretus* (common whitefish): uncultured *Propionibacterium* OTU 591 - done
  - *Coregonus lavaretus* (common whitefish): *Cutibacterium acnes* OTU 1687 - done
  - *Coregonus lavaretus* (common whitefish): uncultured *bacterium* OTU 1751 -

done

- *Solanum melongena* (eggplant): *Moraxella osloensis* OTU 1671
- *Lepisosteus oculatus* (spotted gar): uncultured *Corynebacterium sp.* OTU 1125
- *Lepisosteus oculatus* (spotted gar): *Cutibacterium granulosum* OTU 43
- *Lepisosteus oculatus* (spotted gar): *Cutibacterium granulosum* OTU 1740
- *Lepisosteus oculatus* (spotted gar): uncultured *Corynebacterium sp.* OTU 1128
- *Lepisosteus oculatus* (spotted gar): uncultured *Corynebacterium sp.* OTU 1696
- *Amia calva* (bowfin): *Paracoccus salipaludis* OTU 962
- *Athetis lepigone*: *Pseudomonas putida* OTU 1697
- *Bryum argenteum var. argenteum*: *Pseudomonas putida* OTU 1718 - done
- unidentified marine bacterioplankton: uncultured *Streptococcus sp.* OTU 309

*Results from replication study (Experiment 2)*

*PCR Pooling*

After quality filtering of samples used to assess the requirement for pooling of PCR (**Figure 1**), median read counts were 126552, 110890, and 128873 from PCR reactions in triplicate, duplicate or as a single reaction, respectively from experiment 2. Pairwise Mann-Whitney-U test comparisons showed no significant difference in high-quality read counts generated from reactions in triplicate vs duplicate (p = 0.31), triplicate vs single (p=0.42), or single vs duplicate (p=0.15). We then investigated variation in alpha diversity (measures of the within sample diversity). We did not observe any significant difference between PCR pooling strategies using Kruskall-Wallis tests by Shannon, Simpson, Fisher, Chao1, and Observed indices, and replicates from pair-wise PCR pool conditions showed a strong correlation by Kendall's rank correlation coefficient (**Supplementary Table 1, Supplementary Figure 6**). Beta diversity (measure of the similarity or dissimilarity between two samples) by Bray-Curtis

index clustered by replicate on examination of the PCoA and NMDS ordination plots, and did not significantly differ between PCR pooling strategies by PERMANOVA analysis ($F(2) = 0.47$, $p = 0.94$). The groups did differ by PERMANOVA analysis when compared by sample type i.e. mock vs healthy nasal sample ($F(2) = 35.2$, $p = <0.001$) (**Supplementary Figure 9**). Further, relative abundance of samples by all technical replicates (including various types of mastermix used) appeared to remain similar (**Figure 4 and Figure 5**).

*Mastermix preparation*

After quality filtering of samples used to assess mastermixes for Experiment 2 (**Figure 1**), difference in read counts from samples with manually prepared mastermix (median = 125222) or premixed mastermix (median = 120012) by Mann-Whitney-U test comparison did not reach statistical significance ($p=0.91$). Alpha diversity of replicates from manually prepared or premixed mastermix methods by Shannon, Simpson, Fisher, Chao1, and Observed indices, did not significantly differ between by Mann-Whitney-U comparison (**Supplementary Table 3)**. Beta diversity by Bray-Curtis index clustered by mastermix preparation replicate on examination of the PCoA and NMDS ordination plots **(Supplementary Figure 11)**. Further, relative abundance of samples by all technical replicates (including various types of mastermix used) appeared to remain similar **(Figure 3)**. Replicate numbers in the repeat experiment (Experiment 2) were low and examined with the mock community serially diluted samples alone.

**CARRIAGE Study Team (in alphabetical order)**

Dinesh Aggarwal [1,2], Dr Carl Anderson [2], Katherine L Bellis [1], Beth Blane [1], Joe Brennan [1], Ellena Brooks [1], Susan Burton [3], Dr Adam Butterworth [3], Carol Churcher [1], Professor John Danesh [3], Shannon Duthie [3], Emma Fraser [2], Dr Joan Geoghegan [5], Sophia T Girgis [2], Dr Ewan M Harrison [1,2,3], Rachel Henry [3], Susan Irvine [3], Elisha Johnson [3], Mercedesz Juhasz [3], Stephen Kaptoge [3], Neenu Linson [2], Benjamin McCarthy [2], Dr Amy McMahon [3], Dr Carmel Moore [3], Plamena Naydenova [1], Agnieszka Osmanska [3], Professor Julian Parkhill [4], Professor Sharon Peacock [1], Dr Catherine Perry [3], Dr Kathy E Raven [1], Catarina Ribeiro de Sousa [1], Lauma Sarkane [1], Svetlana Shadrina [3], Dr Matthew R Walker [3].

**Institutions:**

1. Department of Medicine, University of Cambridge
2. Wellcome Sanger Institute
3. Department of Public Health and Primary Care, University of Cambridge
4. Department of Veterinary Medicine, University of Cambridge
5. Institute of Microbiology and Infection, University of Birmingham

**Contributions**

**Funding acquisition:** Dr Carl Anderson [2], Professor John Danesh [2,3], Dr Joan Geoghegan [5], Dr Ewan M Harrison [1,2,3], Professor Julian Parkhill [4], Professor Sharon Peacock [1].

**Leadership and supervision:** Dr Carl Anderson [2], Dr Adam Butterworth [3], Carol Churcher [1], Professor John Danesh [3], Dr Joan Geoghegan [5], Dr Ewan M Harrison [1,2,3], Dr Amy McMahon [3], Dr Carmel Moore [3], Professor Julian Parkhill [4], Professor Sharon Peacock [1]

**Metadata curation:** Katherine L Bellis [1], Beth Blane [1], Joe Brennan [1], Sophia T Girgis [2], Dr Stephen Kaptoge [3], Plamena Naydenova [1], Dr Catherine Perry [3], Dr Kathy E Raven [1], Catarina Ribeiro de Sousa [1], Lauma Sarkane [1], Dr Matthew R Walker [3]

**Project administration:** Katherine L Bellis [1], Beth Blane [1], Ellena Brooks [1], Susan Burton [3], Carol Churcher [1], Shannon Duthie [3], Dr Ewan M Harrison [1,2,3], Rachel Henry [3], Susan Irvine [3], Elisha Johnson [3], Mercedesz Juhasz [3], Dr Amy McMahon [3], Dr Carmel Moore [3]

**Samples and logistics:** Katherine L Bellis [1], Beth Blane [1], Joe Brennan [1], Susan Burton [3], Shannon Duthie [3], Emma Fraser [2] , Sophia T Girgis [2], Dr Ewan M Harrison [1,2,3], Elisha Johnson [3], Mercedesz Juhasz [3], Neenu Linson [2], Benjamin McCarthy [2], Plamena Naydenova [1], Dr Catherine Perry [3], Dr Kathy E Raven [1], Catarina Ribeiro de Sousa [1], Lauma Sarkane [1], Dr Matthew R Walker [3]

**Sequencing and analysis:** Dinesh Aggarwal [2], Katherine L Bellis [1], Dr Adam Butterworth [3], Dr Ewan M Harrison [1,2,3], Dr Stephen Kaptoge [3]

**Data Management:** Dr Catherine Perry [3], Dr Matthew R Walker [3]

**Study Helpdesk**: Susan Burton [3], Shannon Duthie [3], Rachel Henry [3], Susan Irvine [3], Elisha Johnson [3], Mercedesz Juhasz [3], Dr Amy McMahon [3], Dr Carmel Moore [3], Svetlana Shadrina [3], Agnieszka Osmanska [3]

| ENA sample accession | Sample site | Experiment |
|---|---|---|
| ERS15972987 | CARRIAGE | Experiment_1 |
| ERS15972988 | CARRIAGE | Experiment_1 |
| ERS15972932 | CARRIAGE | Experiment_1 |
| ERS15972931 | CARRIAGE | Experiment_1 |
| ERS15972930 | CARRIAGE | Experiment_1 |
| ERS15972933 | CARRIAGE | Experiment_1 |
| ERS15972934 | CARRIAGE | Experiment_1 |
| ERS15972937 | CARRIAGE | Experiment_1 |
| ERS15972935 | CARRIAGE | Experiment_1 |
| ERS15972938 | CARRIAGE | Experiment_1 |
| ERS15972936 | CARRIAGE | Experiment_1 |
| ERS15972940 | CARRIAGE | Experiment_1 |
| ERS15972939 | CARRIAGE | Experiment_1 |
| ERS15972941 | CARRIAGE | Experiment_1 |
| ERS15972944 | CARRIAGE | Experiment_1 |
| ERS15972942 | CARRIAGE | Experiment_1 |
| ERS15972946 | CARRIAGE | Experiment_1 |
| ERS15972943 | CARRIAGE | Experiment_1 |
| ERS15972945 | CARRIAGE | Experiment_1 |
| ERS15972947 | CARRIAGE | Experiment_1 |
| ERS15972948 | Wellcome_Sanger_Institute_Healthy_Volunteers | Experiment_1 |
| ERS15972949 | Wellcome_Sanger_Institute_Healthy_Volunteers | Experiment_1 |
| ERS15972950 | Wellcome_Sanger_Institute_Healthy_Volunteers | Experiment_1 |
| ERS15972951 | Wellcome_Sanger_Institute_Healthy_Volunteers | Experiment_1 |
| ERS15972952 | Wellcome_Sanger_Institute_Healthy_Volunteers | Experiment_1 |
| ERS15972954 | Wellcome_Sanger_Institute_Healthy_Volunteers | Experiment_1 |
| ERS15972953 | Wellcome_Sanger_Institute_Healthy_Volunteers | Experiment_1 |
| ERS15972956 | Wellcome_Sanger_Institute_Healthy_Volunteers | Experiment_1 |
| ERS15972955 | Wellcome_Sanger_Institute_Healthy_Volunteers | Experiment_1 |
| ERS15972957 | Wellcome_Sanger_Institute_Healthy_Volunteers | Experiment_1 |
| ERS15972958 | Control | Experiment_1 |
| ERS15972959 | Control | Experiment_1 |
| ERS15972960 | Control | Experiment_1 |
| ERS15972961 | Control | Experiment_1 |
| ERS15972962 | Mock_Community | Experiment_1 |
| ERS15972963 | Mock_Community | Experiment_1 |
| ERS15972965 | Mock_Community | Experiment_1 |
| ERS15972964 | Mock_Community | Experiment_1 |
| ERS15972967 | Control | Experiment_1 |
| ERS15972966 | Control | Experiment_1 |
| ERS15972968 | CARRIAGE | Experiment_1 |

| | | |
|---|---|---|
| ERS15972969 | CARRIAGE | Experiment_1 |
| ERS15972971 | CARRIAGE | Experiment_1 |
| ERS15972970 | CARRIAGE | Experiment_1 |
| ERS15972974 | CARRIAGE | Experiment_1 |
| ERS15972972 | CARRIAGE | Experiment_1 |
| ERS15972973 | CARRIAGE | Experiment_1 |
| ERS15972975 | CARRIAGE | Experiment_1 |
| ERS15972976 | CARRIAGE | Experiment_1 |
| ERS15972977 | CARRIAGE | Experiment_1 |
| ERS15972978 | CARRIAGE | Experiment_1 |
| ERS15972979 | CARRIAGE | Experiment_1 |
| ERS15972980 | CARRIAGE | Experiment_1 |
| ERS15972981 | CARRIAGE | Experiment_1 |
| ERS15972982 | CARRIAGE | Experiment_1 |
| ERS15972983 | CARRIAGE | Experiment_1 |
| ERS15972984 | CARRIAGE | Experiment_1 |
| ERS15972986 | CARRIAGE | Experiment_1 |
| ERS15972985 | CARRIAGE | Experiment_1 |
| ERS15972989 | CARRIAGE | Experiment_1 |
| ERS15972990 | Mock_Community | Experiment_1 |
| ERS15972991 | Mock_Community | Experiment_1 |
| ERS15972992 | Mock_Community | Experiment_1 |
| ERS15972994 | Mock_Community | Experiment_1 |
| ERS15972993 | Wellcome_Sanger_Institute_Healthy_Volunteers | Experiment_1 |
| ERS15972995 | Wellcome_Sanger_Institute_Healthy_Volunteers | Experiment_1 |
| ERS15972997 | Wellcome_Sanger_Institute_Healthy_Volunteers | Experiment_1 |
| ERS15972996 | Wellcome_Sanger_Institute_Healthy_Volunteers | Experiment_1 |
| ERS15972999 | Wellcome_Sanger_Institute_Healthy_Volunteers | Experiment_1 |
| ERS15972998 | Wellcome_Sanger_Institute_Healthy_Volunteers | Experiment_1 |
| ERS15973000 | Wellcome_Sanger_Institute_Healthy_Volunteers | Experiment_1 |
| ERS15973001 | Wellcome_Sanger_Institute_Healthy_Volunteers | Experiment_1 |
| ERS15973002 | Wellcome_Sanger_Institute_Healthy_Volunteers | Experiment_1 |
| ERS15973003 | Wellcome_Sanger_Institute_Healthy_Volunteers | Experiment_1 |
| ERS15973004 | Mock_Community | Experiment_1 |
| ERS15973005 | Mock_Community | Experiment_1 |
| ERS15973006 | Mock_Community | Experiment_1 |
| ERS15973007 | Mock_Community | Experiment_1 |
| ERS15973009 | Mock_Community | Experiment_1 |
| ERS15973008 | Mock_Community | Experiment_1 |
| ERS15973010 | Wellcome_Sanger_Institute_Healthy_Volunteers | Experiment_1 |
| ERS15973011 | Wellcome_Sanger_Institute_Healthy_Volunteers | Experiment_1 |
| ERS15973012 | Wellcome_Sanger_Institute_Healthy_Volunteers | Experiment_1 |

| | | |
|---|---|---|
| ERS15973013 | Wellcome_Sanger_Institute_Healthy_Volunteers | Experiment_1 |
| ERS15973014 | Wellcome_Sanger_Institute_Healthy_Volunteers | Experiment_1 |
| ERS15973015 | Wellcome_Sanger_Institute_Healthy_Volunteers | Experiment_1 |
| ERS15973016 | Wellcome_Sanger_Institute_Healthy_Volunteers | Experiment_1 |
| ERS15973017 | Wellcome_Sanger_Institute_Healthy_Volunteers | Experiment_1 |
| ERS15973019 | Wellcome_Sanger_Institute_Healthy_Volunteers | Experiment_1 |
| ERS15973018 | Wellcome_Sanger_Institute_Healthy_Volunteers | Experiment_1 |
| ERS15973021 | Mock_Community | Experiment_1 |
| ERS15973020 | Mock_Community | Experiment_1 |
| ERS15973022 | Mock_Community | Experiment_1 |
| ERS15973023 | Mock_Community | Experiment_1 |
| ERS15973024 | Mock_Community | Experiment_1 |
| ERS15973027 | Mock_Community | Experiment_1 |
| ERS15973028 | Wellcome_Sanger_Institute_Healthy_Volunteers | Experiment_2 |
| ERS15973031 | Wellcome_Sanger_Institute_Healthy_Volunteers | Experiment_2 |
| ERS15973034 | Wellcome_Sanger_Institute_Healthy_Volunteers | Experiment_2 |
| ERS15972929 | Control | Experiment_2 |
| ERS15973037 | Wellcome_Sanger_Institute_Healthy_Volunteers | Experiment_2 |
| ERS15973038 | Wellcome_Sanger_Institute_Healthy_Volunteers | Experiment_2 |
| ERS15973039 | Wellcome_Sanger_Institute_Healthy_Volunteers | Experiment_2 |
| ERS15973040 | Wellcome_Sanger_Institute_Healthy_Volunteers | Experiment_2 |
| ERS15973041 | Wellcome_Sanger_Institute_Healthy_Volunteers | Experiment_2 |
| ERS15973042 | Wellcome_Sanger_Institute_Healthy_Volunteers | Experiment_2 |
| ERS15973043 | Wellcome_Sanger_Institute_Healthy_Volunteers | Experiment_2 |
| ERS15973044 | Wellcome_Sanger_Institute_Healthy_Volunteers | Experiment_2 |
| ERS15973045 | Wellcome_Sanger_Institute_Healthy_Volunteers | Experiment_2 |
| ERS15973046 | Mock_Community | Experiment_2 |
| ERS15973047 | Mock_Community | Experiment_2 |
| ERS15973048 | Mock_Community | Experiment_2 |
| ERS15973049 | Mock_Community | Experiment_2 |
| ERS15973051 | Mock_Community | Experiment_2 |
| ERS15973050 | Control | Experiment_2 |
| ERS15973052 | Control | Experiment_2 |
| ERS15973054 | Mock_Community | Experiment_2 |
| ERS15973053 | Mock_Community | Experiment_2 |
| ERS15973055 | Mock_Community | Experiment_2 |
| ERS15973056 | Mock_Community | Experiment_2 |
| ERS15973057 | Control | Experiment_2 |
| ERS15973059 | Mock_Community | Experiment_2 |
| ERS15973058 | Mock_Community | Experiment_2 |
| ERS15973064 | Wellcome_Sanger_Institute_Healthy_Volunteers | Experiment_2 |
| ERS15973065 | Wellcome_Sanger_Institute_Healthy_Volunteers | Experiment_2 |

| | | |
|---|---|---|
| ERS15973066 | Wellcome_Sanger_Institute_Healthy_Volunteers | Experiment_2 |
| ERS15973072 | Mock_Community | Experiment_2 |
| ERS15973070 | Wellcome_Sanger_Institute_Healthy_Volunteers | Experiment_2 |
| ERS15973073 | Wellcome_Sanger_Institute_Healthy_Volunteers | Experiment_2 |
| ERS15973074 | Wellcome_Sanger_Institute_Healthy_Volunteers | Experiment_2 |
| ERS15973075 | Wellcome_Sanger_Institute_Healthy_Volunteers | Experiment_2 |
| ERS15973076 | Wellcome_Sanger_Institute_Healthy_Volunteers | Experiment_2 |
| ERS15973077 | Wellcome_Sanger_Institute_Healthy_Volunteers | Experiment_2 |
| ERS15973078 | Wellcome_Sanger_Institute_Healthy_Volunteers | Experiment_2 |
| ERS15973079 | Wellcome_Sanger_Institute_Healthy_Volunteers | Experiment_2 |
| ERS15973082 | Wellcome_Sanger_Institute_Healthy_Volunteers | Experiment_2 |
| ERS15973084 | Wellcome_Sanger_Institute_Healthy_Volunteers | Experiment_2 |
| ERS15973086 | Wellcome_Sanger_Institute_Healthy_Volunteers | Experiment_2 |
| ERS15973088 | Wellcome_Sanger_Institute_Healthy_Volunteers | Experiment_2 |
| ERS15973092 | Mock_Community | Experiment_2 |
| ERS15973093 | Wellcome_Sanger_Institute_Healthy_Volunteers | Experiment_2 |
| ERS15973094 | Wellcome_Sanger_Institute_Healthy_Volunteers | Experiment_2 |
| ERS15973095 | Wellcome_Sanger_Institute_Healthy_Volunteers | Experiment_2 |
| ERS15973097 | Wellcome_Sanger_Institute_Healthy_Volunteers | Experiment_2 |
| ERS15973096 | Wellcome_Sanger_Institute_Healthy_Volunteers | Experiment_2 |
| ERS15973098 | Wellcome_Sanger_Institute_Healthy_Volunteers | Experiment_2 |
| ERS15973099 | Wellcome_Sanger_Institute_Healthy_Volunteers | Experiment_2 |
| ERS15973100 | Wellcome_Sanger_Institute_Healthy_Volunteers | Experiment_2 |
| ERS15973102 | Wellcome_Sanger_Institute_Healthy_Volunteers | Experiment_2 |
| ERS15973101 | Wellcome_Sanger_Institute_Healthy_Volunteers | Experiment_2 |
| ERS15973104 | Mock_Community | Experiment_2 |
| ERS15973103 | Mock_Community | Experiment_2 |
| ERS15973105 | Mock_Community | Experiment_2 |
| ERS15973107 | Mock_Community | Experiment_2 |
| ERS15973110 | Mock_Community | Experiment_2 |
| ERS15973106 | Mock_Community | Experiment_2 |
| ERS15973108 | Mock_Community | Experiment_2 |
| ERS15973109 | Control | Experiment_2 |

**The STORMS checklist. An editable version for adaptation and inclusion in publications is available from https://stormsmicrobiome.org**

| Number | Item | Recommendation | Item Source | Additional Guidance | Yes/No/NA | Comments or location in manuscript |
|---|---|---|---|---|---|---|
| **Abstract** | | | | | | |
| 1.0 | Structured or Unstructured Abstract | Abstract should include information on background, methods, results, and conclusions in structured or unstructured format. | STORMS | | Yes | (in accordance to journal requirements) |
| 1.1 | Study Design | State study design in abstract. | STORMS | See 3.0 for additional information on study design. | NA | Not required by journal |
| 1.2 | Sequencing methods | State the strategy used for metagenomic classification. | STORMS | For example, targeted 16S by qPCR or sequencing, shotgun metagenomics, metatranscriptomics, etc. | Yes | Abstract |
| 1.3 | Specimens | Describe body site(s) studied. | STORMS | | Yes | Abstract |
| **Introduction** | | | | | | |
| 2.0 | Background and Rationale | Summarize the underlying background, scientific evidence, or theory driving the current hypothesis as well as the study objectives. | STORMS | | Yes | Background |
| 2.1 | Hypotheses | State the pre-specified hypothesis. If the study is exploratory, state any pre-specified study objectives. | STORMS | | Yes | Background |
| **Methods** | | | | | | |
| 3.0 | Study Design | Describe the study design. | STORMS | Observational (Case-Control, Cohort, Cross-sectional survey, etc.) or Experimental (Randomized controlled trial, Non-randomized controlled trial, etc.). For a brief description of common study designs see: DOI: 10.11613/BM.2014.022<br><br>If applicable, describe any blinding (e.g. single or double-blinding) used in the course of the study. | Yes | Methods |

| | | | | | | |
|---|---|---|---|---|---|---|
| 3.1 | Participants | State what the population of interest is, and the method by which participants are sampled from that population. Include relevant information on physiological state of the subjects or stage in the life history of disease under study when participants were sampled. | STORMS | Examples of the population of interest could be: adults with no chronic health conditions, adults with type II diabetes, newborns, etc. This is the total population to whom the study is hoped to be generalizable to. The sampling method describes how potential participants were selected from that population.<br><br>If the participants are from a substudy of a larger study, provide a brief description of that study and cite that study.<br><br>Clearly state how cases and controls are defined.<br><br>An example of relevant physiological state might be pre/post menopausal for a vaginal microbiome study; examples of stage in the life history of disease could be whether specimens were collected during active or dormant disease, or before or after treatment. | Yes | Methods |
| 3.2 | Geographic location | State the geographic region(s) where participants were sampled from. | MIxS: geographic location (country and/or sea,region) | Geographic coordinates can be reported to prevent potential ambiguities if necessary. | Yes | Methods |
| 3.3 | Relevant Dates | State the start and end dates for recruitment, follow-up, and data collection. | STORMS | Recruitment is the period in which participants are recruited for the study. In longitudinal studies, follow-up is the date range in which participants are asked to complete a specific assessment. Finally, data collection is the total period in which data is being collected from participants including during initial recruitment through all follow-ups. | Yes | Methods |

| | | | | | | |
|---|---|---|---|---|---|---|
| 3.4 | Eligibility criteria | List any criteria for inclusion and exclusion of recruited participants. | Modified STROBE | Among potential recruited participants, how were some chosen and others not? This could include criteria such as sex, diet, age, health status, or BMI.<br><br>If there is a primary and validation sample, describe inclusion/exclusion criteria for each. | NA | |
| 3.5 | Antibiotics Usage | List what is known about antibiotics usage before or during sample collection. | STORMS | If participants were excluded due to current or recent antibiotics usage, state this here.<br><br>Other factors (e.g. proton pump inhibitors, probiotics, etc.) that may influence the microbiome should also be described as well. | NA | |
| 3.6 | Analytic sample size | Explain how the final analytic sample size was calculated, including the number of cases and controls if relevant, and reasons for dropout at each stage of the study. This should include the number of individuals in whom microbiome sequencing was attempted and the number in whom microbiome sequencing was successful. | STORMS | Consider use of a flow diagram (see template at https://stormsmicrobiome.org/figures). Also state sample size in abstract.<br><br>If power analysis was used to calculate sample size, describe those calculations. | Yes | Methods |
| 3.7 | Longitudinal Studies | For longitudinal studies, state how many follow-ups were conducted, describe sample size at follow-up by group or condition, and discuss any loss to follow-up. | STORMS | If there is loss to follow-up, discuss the likelihood that drop-out is associated with exposures, treatments, or outcomes of interest. | NA | NA |
| 3.8 | Matching | For matched studies, give matching criteria. | Modified STROBE | "Matched" refers to matching between comparable study participants as cases and controls or exposed / unexposed.<br><br>Indicate whether participants were individual or frequency matched and in what ratio were they matched (e.g. 1 case to 1 control). | NA | NA |
| 3.9 | Ethics | State the name of the institutional review board that approved the study and protocols, protocol number and date of approval, and procedures for obtaining informed consent from participants. | STORMS | | Yes | Methods and Declarations |
| 4.0 | Laboratory methods | State the laboratory/center where laboratory work was done. | STORMS | Provide a reference to complete lab protocols if previously published elsewhere such as on protocols.io. Note any modifications of lab protocols | Yes | Methods |

| | | | | | | |
|---|---|---|---|---|---|---|
| | | | | and the reason for protocol modifications. | | |
| 4.1 | Specimen collection | State the body site(s) sampled from and how specimens were collected. | MIxS: sample collection device or method; host body site | Use terms from the Uber-anatomy Ontology (https://www.ebi.ac.uk/ols/ontologies/uberon) to describe body sites in a standardized format. | Yes | Methods |
| 4.2 | Shipping | Describe how samples were stored and shipped to the laboratory. | STORMS | Include length of time from collection to receipt by the lab and if temperature control was used during shipping. | Yes | Methods |
| 4.3 | Storage | Describe how the laboratory stored samples, including time between collection and storage and any preservation buffers or refrigeration used. | STORMS | State where each procedure or lot of samples was done if not all in the same place.<br><br>Include reagent/lot/catalogue #s for storage buffers. | Yes | Methods |
| 4.4 | DNA extraction | Provide DNA extraction method, including kit and version if relevant. | MIxS: nucleic acid extraction | If any DNA quantification methods were used prior to DNA amplification or at the pooling step of library preparation, state so here. | Yes | Methods |
| 4.5 | Human DNA sequence depletion or microbial DNA enrichment | Describe whether human DNA sequence depletion or enrichment of microbial or viral DNA was performed. | STORMS | | NA | NA |
| 4.6 | Primer selection | Provide primer selection and DNA amplification methods as well as variable region sequenced (if applicable). | MIxS: pcr primers | | Yes | Methods |
| 4.7 | Positive Controls | Describe any positive controls (mock communities) if used. | STORMS | If used, should be deposited under guidance provided in the 8.X items. | Yes | Methods |
| 4.8 | Negative Controls | Describe any negative controls if used. | STORMS | If used, should be deposited under guidance provided in the 8.X items. | Yes | Methods |
| 4.9 | Contaminant mitigation and identification | Provide any laboratory or computational methods used to control for or identify microbiome contamination from the environment, reagents, or laboratory. | STORMS | Includes filtering of reagents and other steps to minimize contamination. It is relevant to state whether the specimens of interest have low microbial load, which makes contamination especially relevant. | Yes | Methods and Results |

| | | | | | | |
|---|---|---|---|---|---|---|
| 4.10 | Replication | Describe any biological or technical replicates included in the sequencing, including which steps were replicated between them. | STORMS | Replication may be biological (redundant biological specimens) or technical (aliquots taken at different stages of analysis) and used in extraction, sequencing, preprocessing, and/or data analysis. | Yes | Methods and Results |
| 4.11 | Sequencing strategy | Major divisions of strategy, such as shotgun or amplicon sequencing. | MIxS: sequencing method | For amplicon sequencing (for example, 16S variable region), state the region selected. State the model of sequencer used. | Yes | Methods |
| 4.12 | Sequencing methods | State whether experimental quantification was used (QMP/cell count based, spike-in based) or whether relative abundance methods were applied. | STORMS | These include read length, sequencing depth per sample (average and minimum), whether reads are paired, and other parameters. | Yes | Methods |
| 4.13 | Batch effects | Detail any blocking or randomization used in study design to avoid confounding of batches with exposures or outcomes. Discuss any likely sources of batch effects, if known. | STORMS | Sources of batch effects include sample collection, storage, library preparation, and sequencing and are commonly unavoidable in all but the smallest of studies. | Yes | Results |
| 4.14 | Metatranscriptomics | Detail whether any mRNA enrichment was performed and whether/how retrotranscription was performed prior to sequencing. Provide size range of isolated transcripts. Describe whether the sequencing library was stranded or not. Provide details on sequencing methods and platforms. | STORMS | Provide details on any internal standards which may have been used as well as parameters and versions of any software or databases used. | NA | NA |
| 4.15 | Metaproteomics | Detail which protease was used for digestion. Provide details on proteomic methods and platforms (e.g. LC-MS/MS, instrument type, column type, mass range, resolution, scan speed, maximum injection time, isolation window, normalised collision energy, and resolution). | STORMS | Provide details on any internal standards which may have been used as well as parameters and versions of any software or databases used. | NA | NA |
| 4.16 | Metabolomics | Specify the analytic method used (such as nuclear magnetic resonance spectroscopy or mass spectrometry). For mass spectrometry, detail which fractions were obtained (polar and/or non-polar) and how these were analyzed. Provide details on metabolomics methods and platforms (e.g. derivatization, instrument type, injection type, column type and instrument settings). | STORMS | Provide details on any internal standards which may have been used as well as parameters and versions of any software or databases used. | NA | NA |

| | | | | | | |
|---|---|---|---|---|---|---|
| 5.0 | Data sources/ measurement | For each non-microbiome variable, including the health condition, intervention, or other variable of interest, state how it was defined, how it was measured or collected, and any transformations applied to the variable prior to analysis. | MIxS: host disease status | State any sources of potential bias in measurements, for example multiple interviewers or measurement instruments, and whether these potential biases were assessed or accounted for in study design.<br><br>Use terms from a standardized ontology such as the Experimental Factor Ontology (https://www.ebi.ac.uk/efo/) to describe variables of interest in a standardized format. | Yes | Methods |
| 6.0 | Research design for causal inference | Discuss any potential for confounding by variables that may influence both the outcome and exposure of interest. State any variables controlled for and the rationale for controlling for them. | STORMS | For causal inference, this item refers to describing the assumptions that would be required to draw causal inferences from observational data. See Vujkovic-Cvijin, I., Sklar, J., Jiang, L. et al. Host variables confound gut microbiota studies of human disease. Nature 587, 448–454 (2020). https://doi.org/10.1038/s41586-020-2881-9 for more details on confounding in observational microbiome studies.<br><br>For example, hypothesized confounders may be controlled for by multivariable adjustment. Consider using a directed acyclic graph (DAG) to describe your causal model and justify any variables controlled for. DAGs can be made using www.dagitty.net. | NA | NA |
| 6.1 | Selection bias | Discuss potential for selection or survival bias. | STORMS | Selection bias can occur when some members of the target study population are more likely to be included in the study/final analytic sample than others. Some examples include survival bias (where part of the target study population is more likely to die before they can be studied), convenience sampling (where members of the target study population are not selected at random), and loss to follow-up (when probability of dropping out is related to one of the things being studied). | NA | NA |

| | | | | | | |
|---|---|---|---|---|---|---|
| 7.0 | Bioinformatic and Statistical Methods | Describe any transformations to quantitative variables used in analyses (e.g. use of percentages instead of counts, normalization, rarefaction, categorization). | STORMS | If a variable is analyzed using different transformations, state rationale for the transformation and for each analyses which version of the variable is used.<br><br>In case of any complex or multistep transformations, give enumerated instructions for reproducing those transformations. | Yes | Methods and Results |
| 7.1 | Quality Control | Describe any methods to identify or filter low quality reads or samples. | MIxS: sequence quality check | If samples were excluded based on quality or read depth, list the criteria used, the number of samples excluded, and the final sample size after quality control. | Yes | Methods |
| 7.2 | Sequence analysis | Describe any taxonomic, functional profiling, or other sequence analysis performed. | MIxS: feature prediction; similarity search method | | Yes | Methods |
| 7.3 | Statistical methods | Describe all statistical methods. | Modified STROBE | Describe any statistical tests used, exploratory data analysis performed, dimension reduction methods/unsupervised analysis, alpha/beta metrics, and/or methods for adjusting for measurement bias.<br><br>If multiple statistical methods are possible, discuss why the methods used were selected.<br><br>If a multiple hypothesis testing correction method was used, describe the type of correction used.<br><br>State which taxonomic levels are analyzed. | Yes | Methods |
| 7.4 | Longitudinal analysis | If the study is longitudinal, include a section that explicitly states what analysis methods were used (if any) to account for grouping of measurements by individual or patterns over time. | STORMS | | NA | NA |
| 7.5 | Subgroup analysis | Describe any methods used to examine subgroups and interactions. | STROBE | | NA | NA |

| | | | | | | |
|---|---|---|---|---|---|---|
| 7.6 | Missing data | Explain how missing data were addressed. | STROBE | "Missing data" refers to participant measurements such as covariates, exposures, outcomes, or time points that should have been collected but were not, not to zeros in taxonomic abundance tables or data points not applicable to that observation. | NA | NA |
| 7.7 | Sensitivity analyses | Describe any sensitivity analyses. | STROBE | | Yes | Methods (mock microbial community in dilutions) |
| 7.8 | Findings | State criteria used to select findings for reporting. | STORMS | For example, false discovery rate with total number of tests, effect size threshold, significance threshold, microbes of interest. | Yes | Methods |
| 7.9 | Software | Cite all software (including read mapping software) and databases (including any used for taxonomic reference or annotating amplicons, if applicable) used. Include version numbers. | Modified STREGA | Installed packages, add-ons or libraries should be stated and cited in addition to the software used.<br><br>All parameters employed that differ from the default of that software/version should be provided.<br><br>This is in addition to, not a replacement for, publishing of code as outlined in the section Reproducible Research. | Yes | Methods |
| 8.0 | Reproducible research | Make a statement about whether and how others can reproduce the reported analysis. | STORMS | Any protected information that has been excluded or provided under controlled access should be listed along with any relevant data access procedures. "On request from authors" is not sufficiently detailed; formal data access procedures and conditions should be defined.<br><br>If data are unavailable, state so clearly.<br><br>Consider using a specialized rubric for reproducible research (such as: https://mbio.asm.org/content/9/3/e00525-18.short). | Yes | Data Availability |

| | | | | | | |
|---|---|---|---|---|---|---|
| | | | | Consider preregistering the study protocol (such as on osf.io or https://plos.org/open-science/preregistration/). | | |
| 8.1 | Raw data access | State where raw data may be accessed including demultiplexing information. | STORMS | Robust, long-term databases such as those hosted by NCBI and EBI are preferred. If using a private repository, provide rationale. | Yes | Data Availability |
| 8.2 | Processed data access | State where processed data may be accessed. | STORMS | Unfiltered data should be provided.<br><br>Robust, long-term databases such as those hosted by NCBI and EBI-EMBL are preferred. Repositories like zenodo (https://zenodo.org/) or publisso (https://www.publisso.de/en/working-for-you/doi-service/) can be used to provide a DOI and long-term storage for processed datasets, even those which cannot be published openly. | NA | NA |
| 8.3 | Participant data access | State where individual participant data such as demographics and other covariates may be accessed, and how they can be matched to the microbiome data. | STORMS | If re-categorized, transformed, or otherwise derived variables were used in the analysis, these variables or code for deriving them should be provided.<br><br>Examples of how participant data can be matched to microbiome data are: using the same set of anonymized identifiers, or using different anonymized identifiers but providing a map. | NA | NA |

| | | | | | | |
|---|---|---|---|---|---|---|
| | | | | Provided data should be sufficient to independently replicate the current analysis. | | |
| 8.4 | Source code access | State where code may be accessed. | STORMS | If a standard or formalized workflow was employed, reference it here. | NA | NA |
| 8.5 | Full results | Provide full results of all analyses, in computer-readable format, in supplementary materials. | STORMS | For example, any fold-changes, p-values, or FDR values calculated, provided as a spreadsheet.<br><br>Use a machine-readable, plain-text format such as csv or tsv. | NA | NA |
| **Results** | | | | | | |
| 9.0 | Descriptive data | Give characteristics of study participants (e.g. dietary, demographic, clinical, social) and information on exposures and potential confounders. | STROBE | Typically reported in a table included in the paper or as a supplementary table. Indicate number of participants with missing data for each variable of interest.<br><br>This includes environmental and lifestyle factors that may affect the relationship between the microbiome and the condition of interest. Participant diet and medication use should be summarized, if known.<br><br>At minimum, age and sex of all participants should be summarized. | Yes | Methods |
| 10.0 | Microbiome data | Report descriptive findings for microbiome analyses with all applicable outcomes and covariates. | STORMS | This includes measures of diversity as well as relative abundances. These descriptive findings should be reported both for the sample overall and for individual groups. | Yes | Results |

| | | | | | | |
|---|---|---|---|---|---|---|
| 10.1 | Taxonomy | Identify taxonomy using standardized taxon classifications that are sufficient to uniquely identify taxa. | STORMS | If not using full taxonomic hierarchy, make sure it is clear whether names stated are species, genera, family, etc.<br><br>Italicize genus/species pairs. Consult journal guidelines or standardized references on taxonomic nomenclature. For instance, https://wwwnc.cdc.gov/eid/page/scientific-nomenclature | Yes | Results |
| 10.2 | Differential abundance | Report results of differential abundance analysis by the variable of interest and (if applicable) by time, clearly indicating the direction of change and total number of taxa tested. | STORMS | If there are more than two groups, include omnibus (multigroup) test results if applicable to the research question.<br><br>If applicable, reported effect sizes should include a measure of uncertainty such as the confidence interval. | Yes | Results |
| 10.3 | Other data types | Report other data analyzed--e.g. metabolic function, functional potential, MAG assembly, and RNAseq. | STORMS | | NA | NA |
| 10.4 | Other statistical analysis | Report any statistical data analysis not covered above. | STORMS | This could include subgroup analysis, sensitivity analyses, and cluster analysis.<br><br>Visualizations should be easily interpretable and colorblind-friendly. The caption and/or main text should provide a detailed description of visualizations for visually-impaired readers. | Yes | Results |
| **Discussion** | | | | | | |
| 11.0 | Key results | Summarise key results with reference to study objectives | STROBE | | Yes | Discussion |

| | | | | | | |
|---|---|---|---|---|---|---|
| 12.0 | Interpretation | Give a cautious overall interpretation of results considering objectives, limitations, multiplicity of analyses, results from similar studies, and other relevant evidence. | STROBE | Define or clarify any subjective terms such as "dominant," "dysbiosis," and similar words used in interpretation of results.<br><br>When interpreting the findings, consider how the interpretation of the findings may be summarized or quoted for the general public such as in press releases or news articles.<br><br>If causal language is used in the interpretation (such as "alters," "affects," "results in," "causes," or "impacts"), assumptions made for causal inference should be explicitly stated as part of 6.0 and 13.0.<br><br>Distinguish between function potential (ie inferred from metagenomics) and observed activity (ie metatranscriptomic, metabolomic, proteomic) if discussing microbial function. | Yes | Discussion |
| 13.0 | Limitations | Discuss limitations of the study, taking into account sources of potential bias or imprecision. | STROBE | Also consider limitations resulting from the methods (especially novel methods), the study design, and the sample size. | Yes | Discussion |
| 13.1 | Bias | Discuss any potential for bias to influence study findings. | STORMS | May include sampling method, representativeness of study participants, or potential confounding. | Yes | Discussion |
| 13.2 | Generalizability | Discuss the generalisability (external validity) of the study results | STROBE | To what populations or other settings do you expect the conclusions to generalize? | Yes | Discussion |
| 14.0 | Ongoing/future work | Describe potential future research or ongoing research based on the study's findings. | STORMS | | Yes | Discussion |
| **Other information** | | | | | | |
| 15.0 | Funding | Give the source of funding and the role of the funders for the present study and, if applicable, for the original study on which the present article is based | STROBE | | Yes | Funding |

| | | | | | | |
|---|---|---|---|---|---|---|
| 15.1 | Acknowledgements | Include acknowledgements of those who contributed to the research but did not meet critera for authorship. | STORMS | For general guidelines on authorship, see http://www.icmje.org and https://www.elsevier.com/authors/journal-authors/policies-and-ethics/credit-author-statement | Yes | Acknowledgements |
| 15.2 | Conflicts of Interest | Include a conflicts of interest statement. | STORMS | | Yes | Competing interests |
| 16.0 | Supplements | Indicate where supplements may be accessed and what materials they contain. | STORMS | | Yes | Supplementary data |
| 17.0 | Supplementary data | Provide supplementary data files of results with for all taxa and all outcome variables analyzed. Indicate the taxonomic level of all taxa. | STORMS | Depending on the analysis performed, examples of the supplemental results included could be mean relative abundance, differential abundance, raw p-value, multiple hypothesis testing-adjusted p-values, and standard error.<br><br>All discussed taxa should include the taxonomic level (e.g. class, order, genus). | Yes | |
